# Room-Temperature Columnar Liquid Crystals from Hydrogen-Bonded Dendritic Assemblies

**DOI:** 10.1021/acsomega.6c04985

**Published:** 2026-08-01

**Authors:** Sivakumar Irla, Velayudhan A. Raghunathan, Sandeep Kumar

**Affiliations:** † 54630 Raman Research Institute, Soft Condensed Matter, C. V. Raman Avenue, Sadashivanagar, Bangalore 560080, India; ‡ Department of Chemistry and Biochemistry, Sharda School of Engineering and Science, Sharda University, Greater Noida 201310, India

## Abstract

Dendrimers represent a distinctive class of synthetic macromolecules with broad utility across soft matter, materials science, nanotechnology, and biomedical sciences. In this study, we describe the synthesis of carboxylic acid-functionalized first and second-generation dendrimers using CuAAC (copper-catalyzed azide, alkyne cycloaddition) click chemistry. Dendrimers exhibit thermotropic mesomorphic behavior and form robust hydrogen-bonded supramolecular complexes with benzotriazole or benzimidazole derivatives. Notably, the hydrogen-bonded complexes also exhibit mesomorphism. Interestingly, some of these exhibit a room-temperature columnar hexagonal mesophase and transition to cubic phases. By underscoring the pivotal role of noncovalent interactions in modulating liquid-crystalline order, this work provides a systematic investigation of the mesomorphic properties of all of the newly synthesized dendrimers and their supramolecular assemblies.

## Graphical abstract

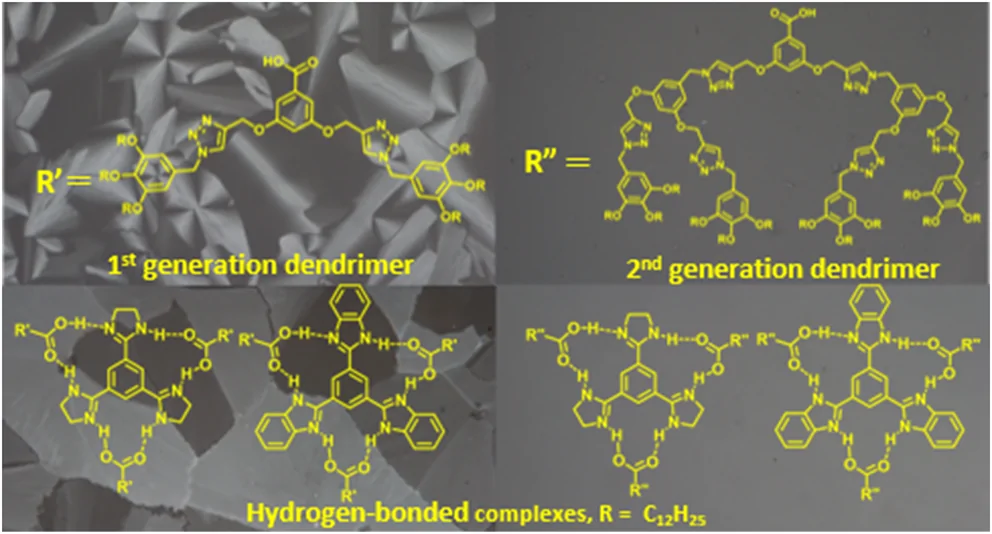

## Introduction

Liquid crystals (LCs) represent a unique state of matter intermediate between conventional liquids and crystalline solids, combining molecular mobility with long-range orientational order to give rise to distinctive optical and electrical properties that have been widely explored for use in display technologies, sensors, and functional materials.^1,2^ The ability to tune mesophase behavior through precise molecular design has made liquid crystals a central focus in both fundamental research and advanced materials science. Dendrimers constitute a unique class of synthetic macromolecules characterized by a highly branched, monodisperse, and three-dimensional architecture.^3–5^ Although widely studied in synthetic polymer chemistry, dendritic motifs are also abundant in nature; for example, the dense array of dendritically branched setae on gecko feet provides exceptional adhesion through maximized surface contact and weak intermolecular interactions.^6^ Inspired by such natural systems, the design and synthesis of dendritic compounds have become an important focus in modern organic chemistry.^7^ Synthetic dendrimers mimic natural branching motifs while offering precisely controlled architectures and high functional-group densities, making them ideal candidates for advanced materials with tailored physical and chemical properties.^8,9^ Since their discovery in the late 1970s, dendrimers have attracted considerable interest in materials science, biomedical sciences, catalysis, sensing, liquid-crystalline systems, and advanced functional materials.^10–13^

Among functional dendrimers, liquid crystal dendrimers (LC dendrimers) have attracted particular attention for their ability to combine molecular order with structural flexibility. Several approaches have been developed to induce liquid-crystalline behavior in dendritic polymers.^13^ The most common methods involve functionalizing dendrimer peripheries with mesogenic or promesogenic units that drive self-assembly into ordered mesophases.^14^ Beyond covalent design, dendrimers and dendrons can also be organized into supramolecular architectures through noncovalent interactions, including van der Waals, ionic, π–π, dipolar, and hydrogen bonding. Microsegregation of incompatible structural units, strongly influenced by branching topology, can likewise generate liquid-crystalline mesophases.^15–20^ Consequently, dendrimers exhibit a wide range of mesophases, including nematic, smectic, columnar, cubic, bicontinuous cubic, and spheroidal cubic phases in both thermotropic and lyotropic systems.^12,21–23^ Cubic phases are broadly classified into two main types: bicontinuous cubic phases, which consist of two interpenetrating continuous networks separated by a curved interface and typically adopt Ia3d space group symmetry, and micellar or columnar cubic phases, which arise from the packing of discrete aggregates into a cubic lattice and most commonly adopt *Pm*3*n* or *Im*3*m* space group symmetry. Both types are optically isotropic under polarizing optical microscopy and can be distinguished by systematic absence analysis of X-ray diffraction data.^23–25^ Cubic phases of *Pm*3*n* symmetry have been widely reported in dendritic and polycatenar systems.^1,14^ Dendritic architectures can also form three-dimensional mesophases such as tetragonal and quasi-liquid-crystalline phases with high rotational symmetry, underscoring their remarkable ability to generate complex, tunable liquid-crystalline structures that are difficult to obtain with conventional low-molecular-weight mesogens.^24,26^

A desirable strategy for inducing or tuning liquid-crystalline behavior in dendritic systems is to exploit noncovalent interactions. Among these, hydrogen bonding has emerged as one of the most versatile tools owing to its directionality, strength, and reversibility. It plays a central role in both chemistry and biology and has been extensively used to induce, stabilize, and modulate mesophases in liquid-crystalline systems.^1,2,27^ Previous studies by Kato and co-workers demonstrated hydrogen-bond -derived smectic and columnar mesophases in folic acid–based systems, while glutamic acid–derived dendritic architectures have shown similarly rich mesomorphic behavior.^28–30^ Donor–acceptor systems, such as carboxylic acids paired with pyridines, benzimidazoles, benzotriazoles, heptazine, and other nitrogen-containing heterocycles, have been widely explored in this context.^31–36^ Such supramolecular approaches enable assembly, reduce synthetic complexity, and enhance stimuli responsiveness. Columnar mesophases formed by disc-shaped molecules are particularly significant owing to their excellent electro-optical properties, making them attractive candidates for optoelectronic applications.^37,38^ Carboxylic acid–functionalized dendrimers are particularly well suited for such complexation, as they engage in strong, directional hydrogen bonding with suitable acceptor molecules, thereby significantly modifying molecular shape, intermolecular interactions, and phase symmetry to tune liquid-crystalline order and thermal stability. Despite these advances, systematic investigations that address the combined influence of dendrimer generation and hydrogen-bond-driven supramolecular assembly on mesomorphic behavior remain scarce.^39^

In parallel, synthetic methodologies for dendrimer construction have evolved significantly.^40^ CuAAC (copper-catalyzed azide–alkyne cycloaddition) click chemistry has emerged as one of the most reliable and efficient routes, enabling precise control over molecular architecture.^41–44^ Dendrimers prepared via CuAAC click chemistry have proven to be robust platforms for liquid-crystalline and supramolecular materials.^45,46^ When combined with peripheral carboxylic acid functionalities, dendrimers synthesized via CuAAC click chemistry become particularly attractive candidates for hydrogen-bonded mesogenic systems. Hydrogen-bonded complexes formed between carboxylic acids and these heterocycles have been reported to exhibit stable liquid-crystalline phases, including nematic, smectic, and columnar ordering.^47–52^ Despite these advantages, the incorporation of carboxylic acid groups into dendritic frameworks and the resulting effects on mesomorphic behavior remain underexplored.

Herein, we report the synthesis of first- and second-generation carboxylic acid–functionalized dendrimers via CuAAC click chemistry and their supramolecular assembly with benzotriazole and benzimidazole derivatives, yielding hydrogen-bonded liquid-crystalline systems. Both dendrimers and their 1:3 hydrogen-bonded complexes exhibit thermotropic mesomorphic behavior, showing the transitions between hexagonal columnar (Col_h_) and cubic (*Pm*3*n*) mesophases. Notably, some supramolecular complexes display stable columnar mesophases at room temperature. The insights gained from this study provide valuable guidelines for the rational design of functional dendritic liquid crystals and supramolecular soft materials for advanced applications in optoelectronics, sensing, and responsive systems.

## Results and discussion

Design and synthesis of a novel class of carboxylic acid-functionalized dendrimers, like first and second generations, and hydrogen-bonded complexes were shown in Scheme S1. The dendrimers were prepared by incorporating two (5-(azidomethyl)-­1,2,3-tris­(dodecyl­oxy)­benzene) units onto a central resorcinol core via CuAAC click chemistry.^53^ Benzotri (imidazole) derivatives (compounds **12** and **13**) were then prepared according to the literature procedures.^54,55^ All compounds were purified by column chromatography on silica gel (100–200 mesh), followed by repeated reprecipitation using suitable analytical-grade solvents. The chemical structures of all compounds were confirmed by ^1^H NMR, ^13^C NMR spectroscopy, and elemental analysis. The detailed synthetic scheme, procedures, and characterization data are given in the Supporting Information. The hydrogen-bonded LC complexes were prepared by dissolving the carboxylic acid derivatives and benzotri (imidazole) derivatives in MeOH/CHCl_3_ and stirring for 2 h, followed by solvent removal. Spectroscopic analysis (FT-IR and ^1^H NMR, ^13^C NMR) and thermal analysis (DSC) confirmed the formation of the supramolecular complexes. The synthesized dendrimers exhibit thermotropic mesomorphic behavior, displaying the columnar hexagonal (Col_h_) and cubic (*Pm*3*n*) phases. Notably, the hydrogen-bonded complexes exhibit a stable hexagonal columnar mesophase at room temperature, which transforms into a cubic (*Pm*3*n*) phase at elevated temperatures.

The structure of all dendrimers (**7, 9, 10, 11)** and their corresponding hydrogen-bonded (**14–17**) liquid crystalline (LC) materials are shown in Scheme 1, and the thermal stability of all LC compounds was evaluated by thermogravimetric analysis (TGA). Each sample was subjected to a heating scan at 10 °C min^–1^ under a nitrogen atmosphere to assess the compound’s thermal stability. All compounds exhibit thermal stability, showing no significant weight loss up to approximately 335 °C. Beyond this temperature, gradual decomposition occurred between 350 and 475 °C, with major degradation completed by around 520 °C. The thermal stability of all the compounds is much higher than their isotropic (Iso) temperature. This indicates that all compounds have excellent thermal stability. The TGA thermograms of dendrimers and hydrogen-bonded complexes are given in Figure 1.

**Figure 1:**
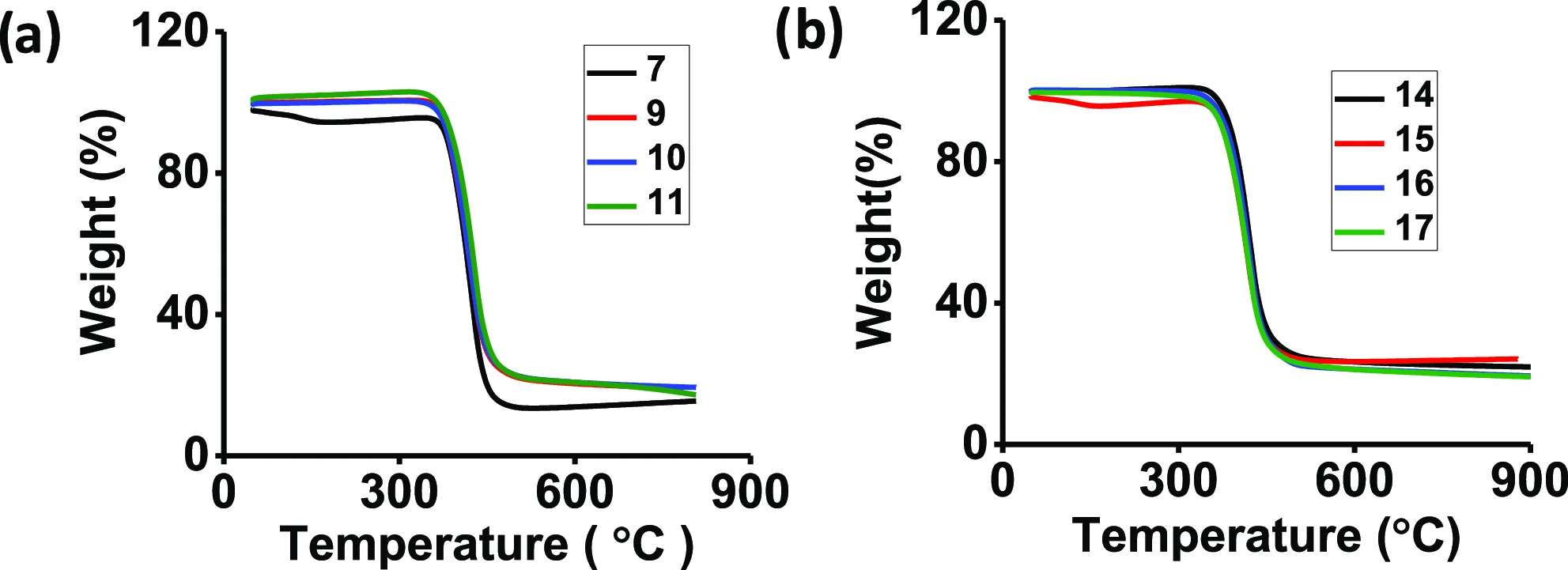
(a) TGA thermograms of liquid-crystalline dendrimers, (b) TGA thermograms of hydrogen-bonded (HB) complexes.

**Scheme 1:**
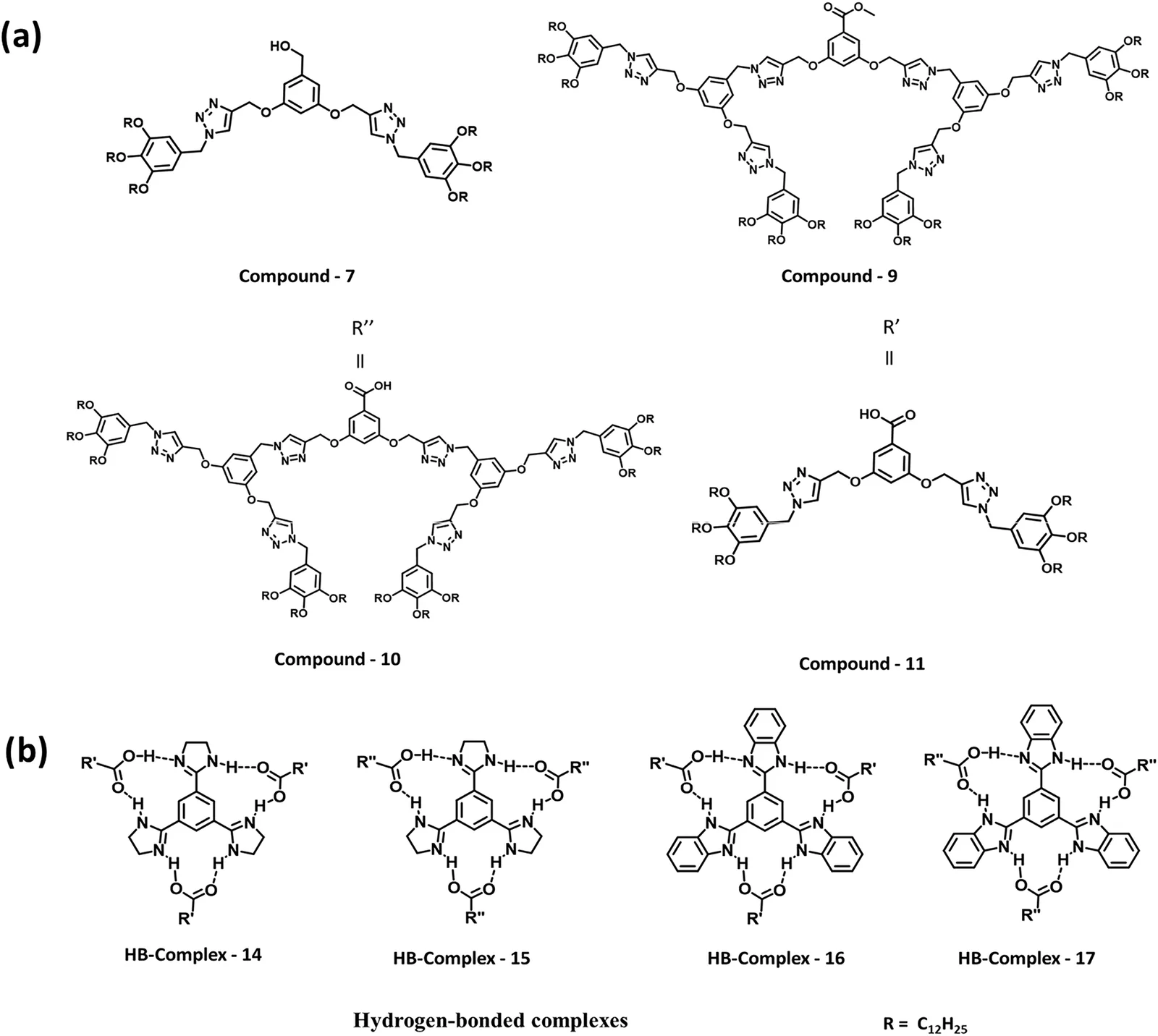
(a) Liquid Crystalline Dendrimers; (b) Hydrogen-Bonded (HB) Complexes

The liquid-crystalline (LC) behavior of the dendrimers was investigated using polarizing optical microscopy (POM), differential scanning calorimetry (DSC), and small-angle X-ray diffraction (SAXS). All the compounds exhibited enantiotropic mesophase behavior. POM textures (Figure 2) recorded upon cooling from the isotropic (Iso) phase revealed focal-conic patterning for compounds **7** and **11**, characteristic of columnar hexagonal (Col_h_) mesophase. In contrast, Compounds **9** and **10** exhibit optically isotropic textures characteristic of the cubic mesophase. DSC data of **7, 9, 10**, and **11** show the enantiotropic mesophase between a high-temperature isotropic (Iso) phase and a low-temperature crystalline (Cr) phase.

**Figure 2:**
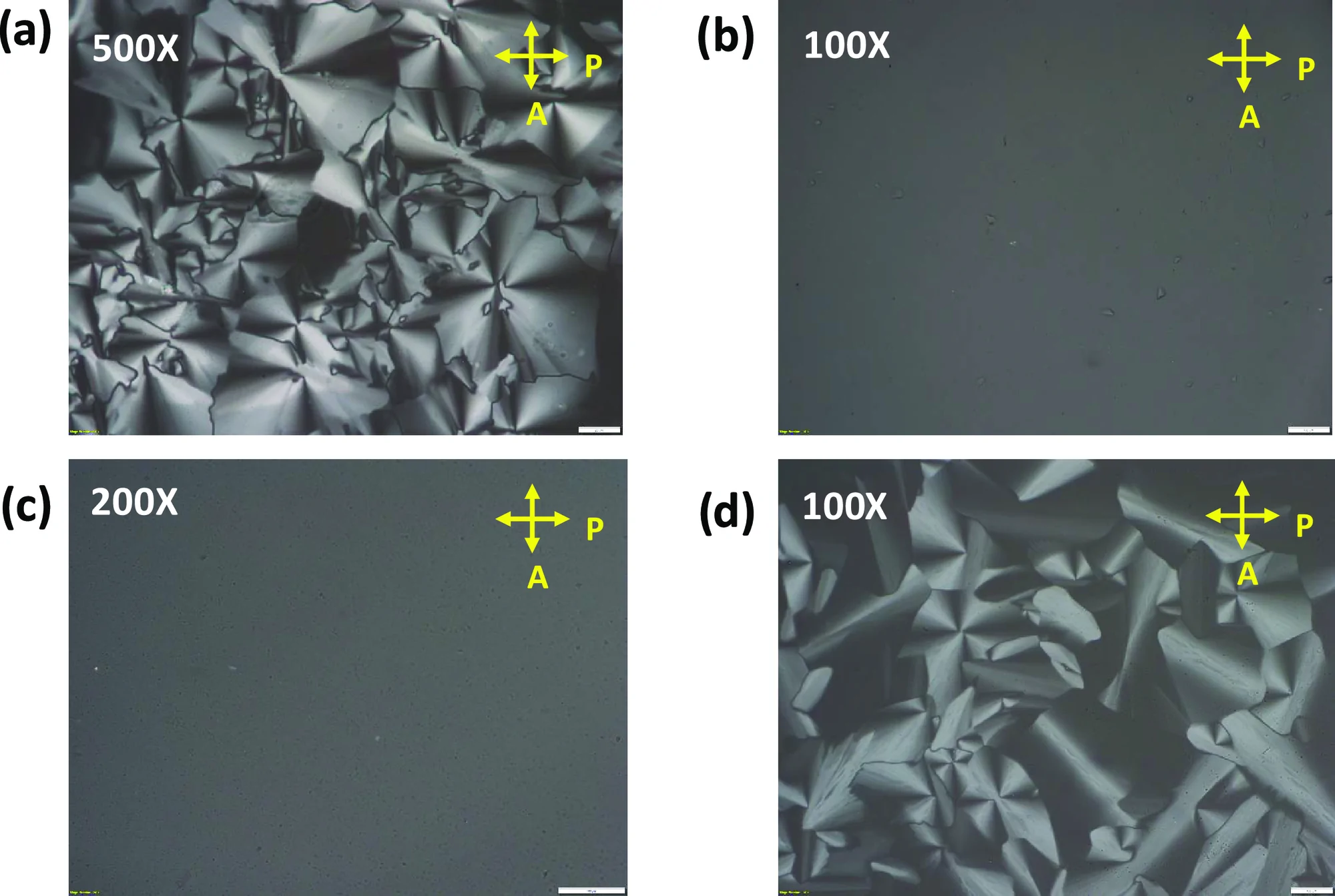
Birefringence texture observed under POM for (a) Compound **7** at 95 °C, (b) Compound **9** at 80 °C, (c) Compound **10** at 135 °C, (d) Compound **11** at 78 °C, at different temperatures upon cooling from the isotropic (Iso) phase.

Compounds **7** and **11** show two transition peaks corresponding to the single Col_h_ mesophase. Compound **7** exhibits a transition from the crystalline (Cr) phase to the columnar hexagonal (Col_h_) mesophase at 61.9 °C, and becomes isotropic (Iso) at 81.7 °C. Upon cooling from the isotropic (Iso) phase, the Col_h_ mesophase reappears at 78.7 °C, followed by crystallization. Compound **11** similarly exhibits a Cr to Col_h_ transition at 38.4 °C and becomes isotropic at 98.2 °C. Upon cooling, the Col_h_ mesophase reappears at 94.4 °C, followed by crystallization.

In contrast, compounds **9** and **10** exhibit two transition peaks corresponding to the single- cubic mesophase, which belongs to the *Pm*3*n* space group. Compound **9** transitions from Cr to Colc at 75.1 °C and becomes isotropic (Iso) at 98.7 °C. Upon cooling from the isotropic (Iso) phase, the cubic (*Pm*3*n*) mesophase reappears at 83.1 °C. Compound **10** transitions from Cr to cubic (*Pm*3*n*) at 97.7 °C and becomes isotropic at 159.5 °C. Upon cooling, the cubic (*Pm*3*n*) mesophase reappears at 156.0 °C, followed by crystallization. All DSC thermograms are shown in Figure 3, and the phase transition temperatures and enthalpy values are presented in Table 1

**Table 1:** Phase Transition Temperatures (°C), Enthalpy Changes, [J/g] of Dendrimers and Hydrogen-Bonded Complexes at the Time of Heating and Cooling Obtained from DSC^a^

compound	heating	cooling
**7**	Cr 61.9 [20.77] Col_h_ 81.7 [1.90] Iso	Iso 78.7 [−1.25] Col_h_
**9**	Cr 75.1 [10.40] cubic (*Pm*3*n*) 98.7 [10.92] Iso	Iso 83.1 [−0.33] cubic (*Pm*3*n*)
**10**	Cr 97.7 [22.49] cubic (*Pm*3*n*) 159.5 [1.10] Iso	Iso 156.0 [−0.66] cubic (*Pm*3*n*)
**11**	Cr 38.4 [1.42] Col_h_ 98.2 [1.04] Iso	Iso 94.4 [−1.04] Col_h_

a Cr–Crystalline phase; Col_h_–Columnar hexagonal phase; cubic (*Pm*3*n*) phase; Iso–Isotropic.

**Figure 3:**
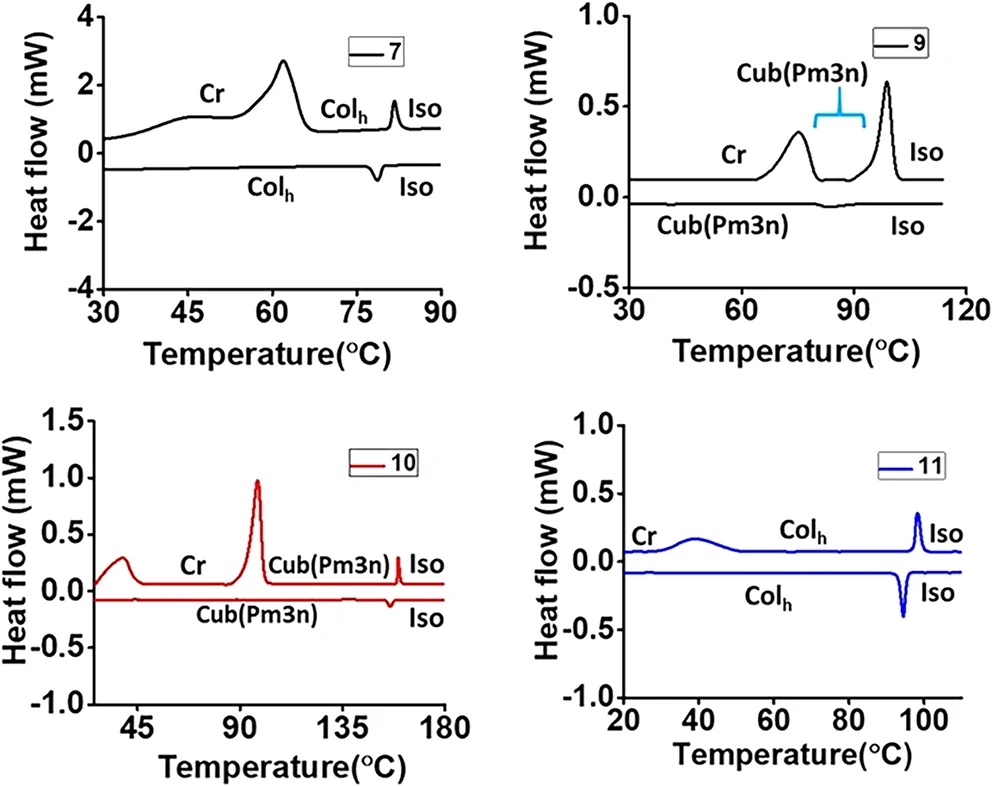
DSC thermograms obtained for the **7**, **9**, **10**, and **11** derivatives showing phase transitions during heating and cooling cycles at the scan rate of 10 °C min^–1^.

To further confirm the supramolecular assembly of **7, 9, 10**, and **11**, small-angle X-ray scattering (SAXS) experiments were carried out. The X-ray diffractograms of all the compounds are shown in Figure 4. Compounds **7** and **11** are found to exhibit the hexagonal columnar (Col_h_) phase upon cooling from the isotropic (Iso) phase, as evidenced by sharp diffraction peaks in the small-angle region, whose spacings (d) are in the ratio 1:1/√3:1/2. In the wide-angle region, the diffractograms exhibit broad peaks at *d* = 4.3–4.50 Å, corresponding to the molten alkyl chains. The lattice parameter (a) is 38.85 Å and 42.37 Å for **7** and **11**, respectively. From the lattice parameter, the number of molecules occupying each slice of a column is estimated to be 2. The X-ray diffractograms of compound **9** at 30 °C and compound **10** at 135 °C show multiple peaks in the small-angle region. These can be indexed on a *Pm*3*n* cubic lattice, and the lattice parameters (a) are estimated to be 84.44 Å and 86.84 Å, respectively. For compounds **9** and **10**, the number of molecules per unit cell in the cubic lattice is 104 and 114, respectively. The *d*-spacings of these compounds and their Miller indices are given in Table 3, along with the lattice parameters.

**Figure 4:**
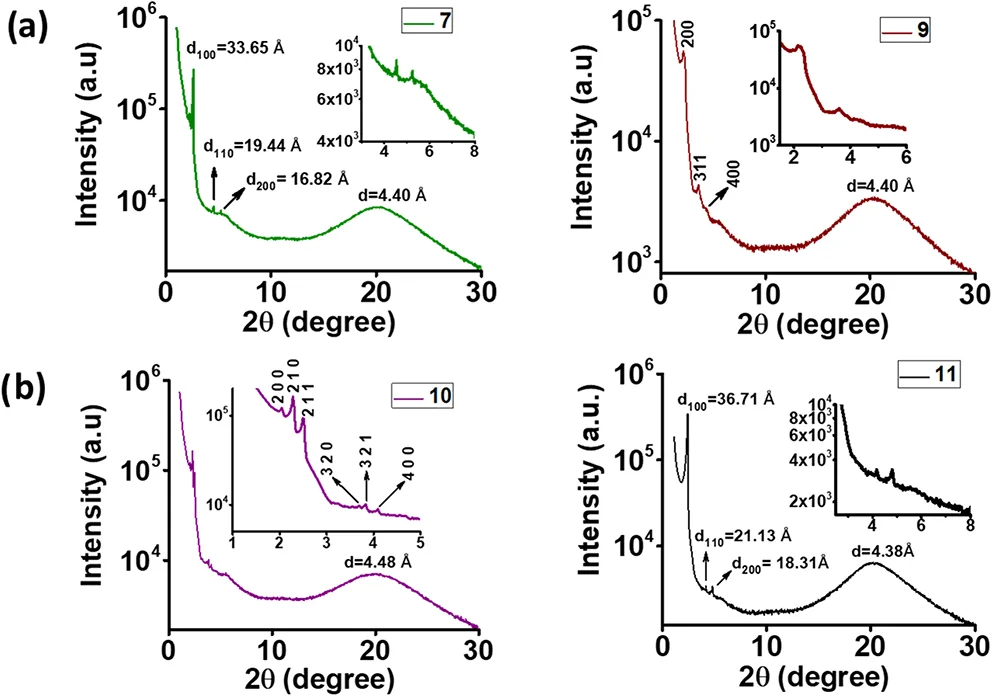
(a) X-ray diffraction patterns of the hexagonal phase of compound **7** at 65 °C (on heating), and the cubic phase of compound **9** at 30 °C (on cooling from the isotropic phase), (b) X-ray diffraction patterns of the hexagonal phase of compound **11** at 25 °C (on cooling from the isotropic phase), and cubic phase of compound **10** at 135 °C (on heating).

### Detailed study of the hydrogen-bonded complexes

Hydrogen-bonded complexes were prepared using 1:3 ratios of benzotriimidazole derivatives to first and second-generation carboxylic acid-functionalized dendrimers. Complex **14** was prepared by dissolving compound **12** with **10**, and complex **15** by dissolving compound **12** with dendrimer **11**, both in MeOH/CHCl3 (1:4 v/v), stirring for 2 h at room temperature, and removing the solvents under reduced pressure. Complex **16** was prepared by dissolving compound **13** with **10**, and Complex **17** by dissolving compound **13** with **11**, both in MeOH/CHCl_3_ (2:3 v/v), followed by stirring for 3 h at 60 °C and subsequent solvent removal. The chemical structure of dendrimers, benzotriimidazole derivatives, the resulting hydrogen-bonded complexes, and schematic representations are shown in Scheme S1. The formation of the hydrogen-bonded complexes was confirmed by FT-IR and NMR spectroscopy along with thermalanalysis. The detailed synthetic scheme, procedures, and full characterization data are provided in the Supporting Information. All complexes exhibit enantiotropic liquid crystalline behavior over a wide temperature range, reflecting the strong, stable hydrogen-bonding interaction between compounds **12** or **13** and dendrimers **10** or **11**.

The formation of the hydrogen-bonded complexes was confirmed by using FTIR. Notably, the infrared spectra of the obtained H-bonded complexes are not simple superpositions of the spectra of the individual components, indicating the presence of specific intermolecular interactions. The FTIR spectra of **11, 12**, and **13**, and their corresponding H-bonded complexes **14** and **16**, are shown in Figure 5, while the spectra of compounds **10, 12**, and **13** and their hydrogen-bonded complexes **15** and **17** are presented in Figure S1. The N–H stretching band of the **12** (benzotriimadazole) derivative is observed at 3134 cm^–1^. Upon complexation, this band becomes significantly weaker and shifts to higher wavenumbers (3238 cm-1), clearly reflecting its involvement in hydrogen bonding. Pronounced changes are also observed in the carbonyl stretching region. In the free carboxylic acid dendrimers, the C=O stretching band at 1696 cm^–1^ corresponds to the hydrogen-bonded dimeric form. After complexation with compound **12**, this dimeric C=O band completely disappears, and a new broadened C=O stretching band appears at 1639 cm^–1^, shifted to lower wavenumbers, consistent with the formation of intermolecular hydrogen bonds between compounds **11** and **12**. Similar spectral changes are observed for the remaining hydrogen-bonded complexes, further confirming the establishment of strong hydrogen-bonding interactions.

**Figure 5:**
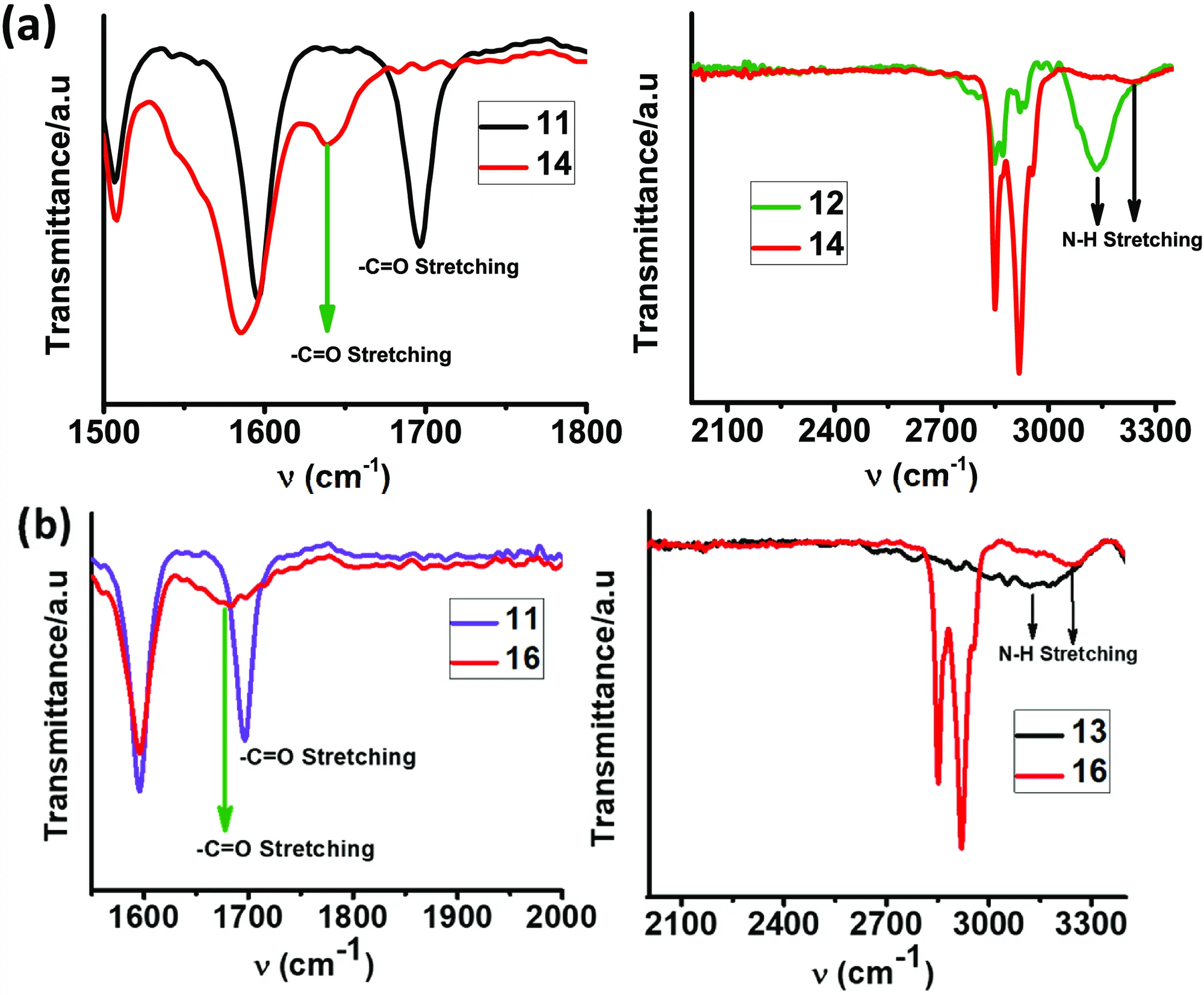
FTIR spectra: comparison in the – NH and C=O stretching region. (a) Pure components of 11 and 12, and their H-bonded complex of **14**. (b) Pure components of 11 and 13, and their H-bonded complex of **16**.

The selectivity of hydrogen bonding in the complexes was further confirmed by the complete disappearance of the homodimeric C=O band around 1700 cm^–1^ upon complexation, confirming that carboxylic acid homodimerization is completely replaced by selective heterodimer formation with the benzotriazole or benzimidazole units. The triazole rings introduced by CuAAC click chemistry are not expected to compete significantly as hydrogen-bond acceptors, as the triazole nitrogens are weaker hydrogen-bond acceptors than benzotriazole or benzimidazole nitrogens due to their lower basicity.^56^ This confirms that hydrogen bonding occurs selectively between the carboxylic acid groups of the dendrimers and the benzotriazole or benzimidazole units rather than with the triazole rings or through homodimerization. The characteristic C=O and N–H stretching frequencies of compounds **10–13** and all hydrogen-bonded complexes (**14–17**) are summarized in Table S1.

Further evidence for hydrogen-bond formation between the benotriimadazole derivatives and the first- and second-generation dendrimers was obtained from ^1^H NMR spectroscopy. The NMR spectra of hydrogen-bonded complexes **14** and **15** were recorded in CDCl_3_, whereas those of complexes **16** and **17** were recorded in CDCl_3_ and in a 1:1 mixture of CDCl_3_ and DMSO-*d*_6,_ respectively. Comparative NMR analyses of individual compounds and their H-bonded complexes of **14** and **15** are given in Figures 6 and S2. Integration of the characteristic proton signals confirms the expected stoichiometry of the supramolecular complexes, consistent with the designed hydrogen-bonded assemblies between the dendrimers and benzotriazole or benzimidazole derivatives. In addition, clear chemical shift changes are observed for the aromatic (Ha) and methylene (Hb) protons of the benzotriazole or benzimidazole units upon complexation. Direct observation of the N–H and COOH protons is not feasible under these conditions, as these protons undergo significant broadening due to strong intermolecular hydrogen bonding interactions and fast dynamic exchange within the supramolecular complex; therefore, hydrogen bonding is inferred from the downfield shifts of neighboring aromatic and methylene protons.

**Figure 6:**
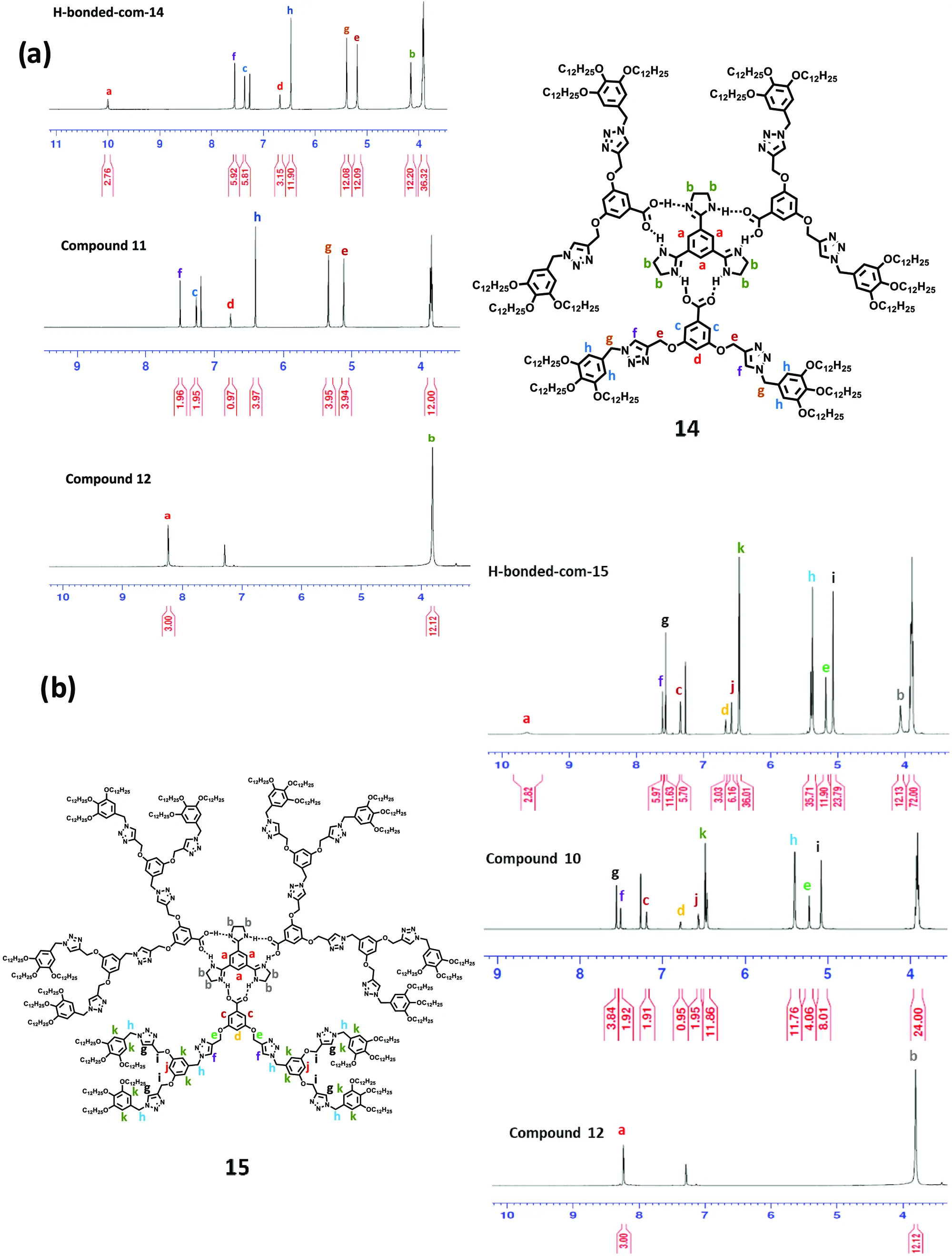
(a) Comparative ^1^H NMR studies of pure compounds **11** and **12**, and the hydrogen complex **14**. (b) Comparative ^1^H NMR studies of pure compounds **10** and **12**, and the hydrogen complex **15**. (a) Comparative ^1^H NMR studies of **11, and 12** pure compounds and, hydrogen complex of **14** (b) Comparative ^1^H NMR studies of **10, and 12** pure compounds and, hydrogen complex of **15**.

In the free benzotriazole derivative **12**, the Ha and Hb protons appear at 8.23 and 3.80 ppm, respectively. Upon formation of the hydrogen-bonded complex **14**, these signals shift downfield to 9.99 and 4.15 ppm, respectively, reflecting deshielding caused by strong intermolecular hydrogen bonding. Similar downfield shifts are observed for the corresponding protons in complex **15**. Comparable spectral changes are also detected for complexes **16** and **17**, with minor variations attributable to differences in dendrimer generation and solvent environment. Overall, the consistent downfield shifts and maintained proton integration ratios across all complexes provide strong ^1^H NMR evidence for the formation of stable hydrogen-bonded supramolecular assemblies, in good agreement with previously reported systems.

Further evidence for hydrogen bond formation was obtained from ^13^C NMR spectroscopy. In the ^13^C NMR spectrum of the free first-generation dendrimer 11, the carbonyl carbon of the carboxylic acid group appears at 170.08 ppm. Upon formation of the hydrogen-bonded complexes, this signal shifts downfield to 172.83 ppm in complex 14 and 172.12 ppm in complex 16. consistent with the involvement of the carbonyl group in intermolecular hydrogen bonding with the benzotriazole or benzimidazole units. For the second-generation dendrimer **10**, the carbonyl carbon signal is not observed in the free dendrimer, which is commonly encountered for high molecular weight dendrimers due to the low relative concentration of the carbonyl carbon, its quaternary nature, and long relaxation times. Notably, upon complexation with benzotriazole or benzimidazole derivatives, the carbonyl carbon becomes clearly visible at ∼170 ppm in complexes **15** and **17**, providing strong additional evidence for the formation of hydrogen-bonded assemblies. These observations are fully consistent with the FTIR and ^1^H NMR data.

The H-bonded supramolecular complexes with liquid-crystalline properties were analyzed using differential scanning calorimetry (DSC), polarizing optical microscopy (POM), and X-ray diffraction (XRD). Compounds **14–17** were all found to be mesomorphic. Compounds **14** and **16** display two distinct mesophases: columnar hexagonal (Col_h_) and cubic (*Pm*3*n*). Whereas complexes **15** and **17** exhibit only the cubic (*Pm*3*n*) mesophase. DSC thermograms (Figure 7) were used to investigate the mesophase behavior of all complexes. Compounds **14** and **16** clearly show three transition peaks associated with the two mesophases. Both complexes exhibit the columnar hexagonal (Col_h_) mesophase at room temperature, and the transition temperatures are 97.84 and 112.52 °C, which become isotropic (Iso) at 193.22 and 176.17 °C, respectively. Upon cooling from the isotropic (Iso) phase, cubic (*Pm*3*n*) mesophase appears at 190.19 °C for **14** and 168.90 °C for **16**. Further cooling results in the re-emergence of the columnar hexagonal (Col_h_) mesophase at 69.46 and 96.82 °C, followed by crystallization (Cr) below −10 °C. Compounds **15** and **17** exhibit two transition peaks corresponding to the single mesophase. Both show broad melting temperatures at 60.55 and 0.41 °C, respectively, and become isotropic (Iso) at 231.27 and 191.18 °C, respectively. Upon cooling from the isotropic (Iso) phase, the cubic (*Pm*3*n*) phase appeared at 224.95 and 181.38 °C, respectively, and remained unaltered to room temperature. Additionally, Compound 17 shows a sharp transition on heating at 0.4 °C corresponding to the melting of the crystalline phase, followed by a broad transition at 55.37 °C attributed to a crystal-to-crystal (Cr → Cr′) transition, reproducibly observed across multiple heating runs, before entering the cubic (*Pm*3*n*) phase. The cubic (*Pm*3*n*) – Iso clearing transition appears at 191.1 °C as a single, well-resolved peak. The observed phase transition temperatures and corresponding enthalpy values are summarized in Table 2.

**Table 2:** Phase Transition Temperatures (°C), Enthalpy Changes, [J/g] of Dendrimers and Hydrogen-Bonded Complexes at the Time of Heating and Cooling Obtained from DSC^a^

compound	heating	cooling
**14**	Cr −10.8 [3.89] Col_h_ 97.8 [0.80] Cubic (*Pm*3*n*) 193.2 [0.46] Iso	Iso 190.1 [−0.35] Cubic (*Pm*3*n*) 69.4 [−1.45] Col_h_ −14.8 [−3.42] Cr
**15**	Cr 60.5 [18.87] Cubic (*Pm*3*n*) 231.2 [2.47] Iso	Iso 224.9 [−0.76] Cubic (*Pm*3*n*)
**16**	Cr −5.6 [4.51] Col_h_ 112.5 [8.77] Cubic (*Pm*3*n*) 176.1 [0.73] Iso	Iso 168.9 [−0.74] Cubic (*Pm*3*n*) 96.8 [−6.51] Col_h_ −11.6 [−4.36] Cr
**17**	Cr −0.41 [5.40] Cr′ 55.37 [4.28] cubic (*Pm*3*n*) 191.1 [4.40] Iso	Iso 181.3 [−1.07] Cubic (*Pm*3*n*) −20.8 [−1.18] Cr

a Cr–Crystalline phase; Cr’-Crystal, Col_h_–Columnar hexagonal phase; cubic (*Pm*3*n*); Iso–Isotropic.

**Figure 7:**
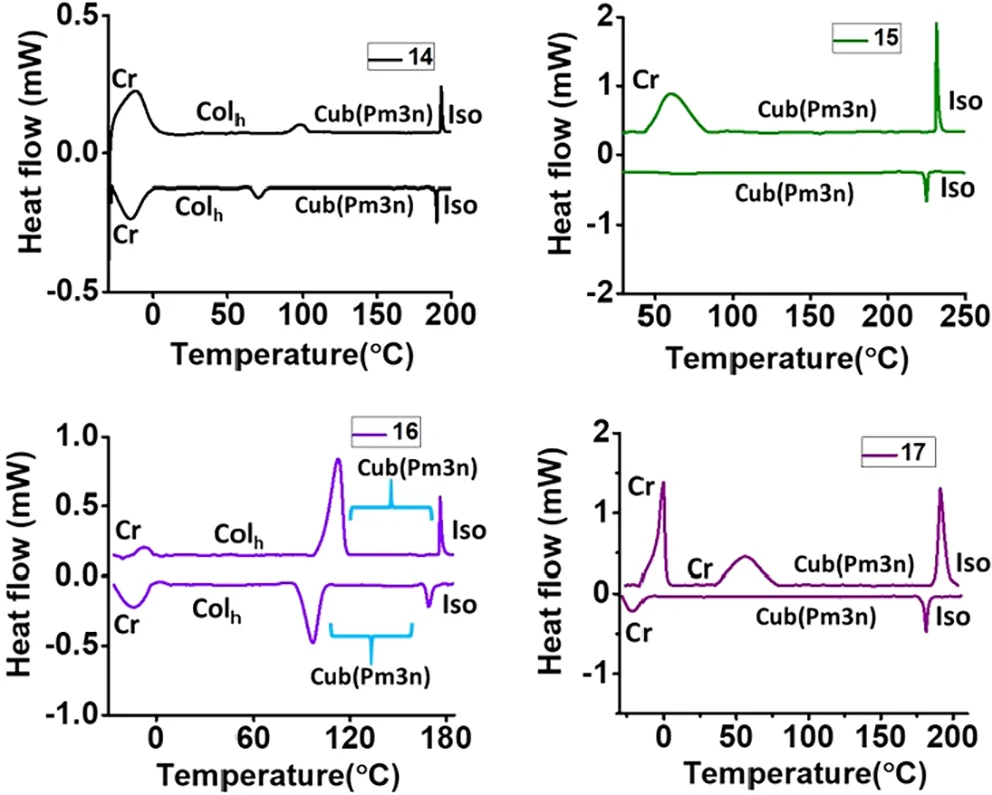
DSC thermograms obtained for the **14**, **15**, **16**, and **17** derivatives showing phase transitions during heating and cooling cycles at a scan rate of 10 °C min^–1^.

The transition temperatures and enthalpies of H-bonded complexes were investigated using DSC and further validated by POM. Liquid-crystalline textures were examined under crossed polarizers upon cooling from the isotropic (Iso) phase. Complexes **14** and **16** exhibit two distinct mesophases. Upon cooling, both display the optically isotropic texture over the ranges 190 to 69 °C and 168 to 96 °C, respectively, characteristic of the cubic (*Pm*3*n*) mesophase. Further cooling leads to the appearance of plate-like textures corresponding to the columnar hexagonal (Col_h_) mesophase. In contrast, Compounds 15 and 17 show only an optically isotropic texture upon cooling from the isotropic (Iso) phase, indicative of a single cubic (*Pm*3*n*) mesophase. All the POM textures are shown in Figure 8. Repeated heating–cooling cycles revealed no evidence of phase segregation, demonstrating that the supramolecular complexes remain intact and stable, likely due to strong hydrogen-bonding interactions.

**Figure 8:**
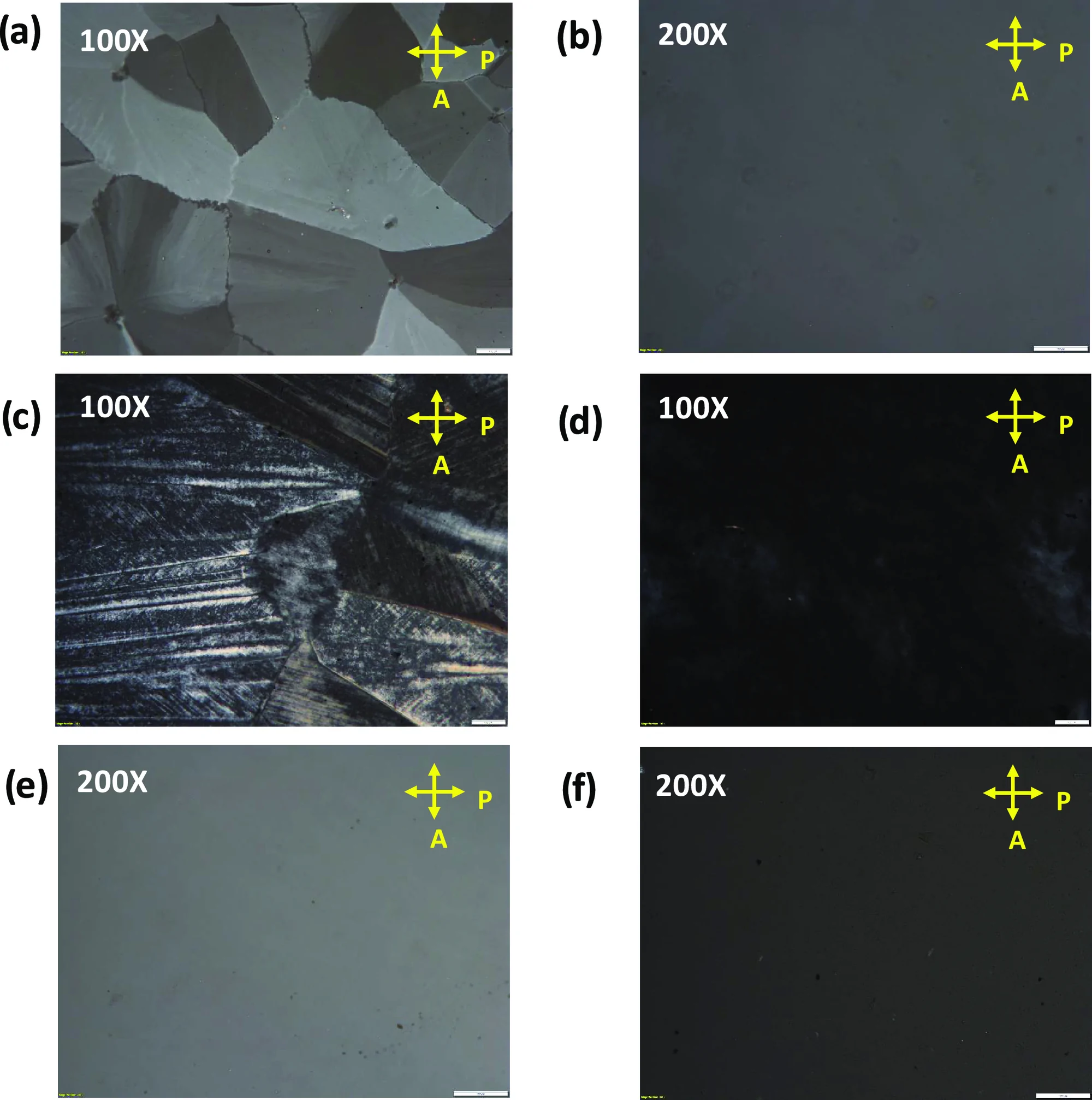
Birefringence texture observed under POM for a compound: (a, b) Compound **14** at 80 and 130 °C, (c, d) Compound **16** at 90 and 140 °C, (e) Compound **15** at 140 °C, (f) Compound **17** at 150 °C at different temperatures upon cooling from the isotropic (Iso) phase.

The liquid crystalline phase behavior of the hydrogen- bonded complexes (**14**, **15**, **16**, and **17**) was examined using X-ray diffraction (XRD) upon cooling from the isotropic (Iso) phase. The X-ray diffraction patterns of the H-bonded complexes of **14** and **16** are shown in Figure 9. Consistent with the DSC and POM analysis, these complexes exhibit two distinct mesophases. Accordingly, X-ray diffraction studies were carried out at temperatures corresponding to the two mesophases. Compounds**14** exhibits four sharp peaks at 50 °C, and compound **16** exhibits six sharp peaks at 28 °C in the small-angle region. Their spacings are in the ratio of 1:1/√3:​1/2:1/√7:​1/√9:1/√13, corresponding to the columnar hexagonal (Col_h_) mesophase. The lattice parameter (a) is found to be 48.57 Å and 51.57 Å, respectively. The wide-angle region exhibits two broad peaks, one at around 4.3 Å corresponding to the molten alkyl chain. Additionally, core–core peaks observed at 3.44 Å and 3.57 Å for compounds 14 and 16, respectively, indicate π–π stacking interactions between the aromatic units in the dendritic framework and benzotriazole or benzimidazole derivatives. Such interactions are well-known to contribute to the stability of columnar mesophases.^37,38^ Furthermore, the broad peaks at 4.3–4.5 Å, corresponding to molten dodecyloxy (C_12_H_25_) alkyl chains, suggest that London dispersion forces (LDF) from the large number of peripheral chains also contribute to mesophase stabilization through microsegregation between the rigid aromatic core and the flexible alkyl periphery.^1,12^ All the sharp peaks in the small-angle region can be indexed on the two-dimensional hexagonal lattice. The number of molecules per slice of the column (Z) was calculated from the XRD data and was found to be very close to 1. At elevated temperatures, both the compounds **14** and **16** show a few sharp peaks in the small-angle region, with their *d*-spacings in the ratio of 2:√10:√20 and 2:√5:​√6:√14:​√16:√30, respectively, that corresponds to the (2 0 0), (3 1 0), (4 2 0) and (2 0 0), (2 1 0), (2 1 1), (3 2 1), (4 0 0), (5 2 1) reflections from a cubic lattice belonging to the space group *Pm*3*n*. The broad peak shown at 4.5 Å in the wide-angle region corresponds to the molten alkyl chains. The lattice parameters (a) of these compounds are 75.38 and 81.12. The number of molecules in the unit cell of the cubic lattice is 51 and 81. The *d*-spacings of these compounds and their Miller indices are given in Table 3, along with the lattice parameters.

**Table 3:** Layer Spacings Obtained from XRD Patterns for **7, 11, 14**, and **16^a^**

compounds	*T* (°C)	*d*-spacing(Å)	phase	parameters
**7**	65	33.65 (1 0 0), 19.44 (1 1 0), 16.82 (2 0 0) 4.41	Col_h_	*a* = 38.85 Å
				*S*_h_ = 1307.44 Å^2^
				*V*_h_ = 5765.84 Å^3^
				*Z* = 2.1
**9**	30	42.22 (2 0 0), 25.15 (3 1 1), 20.94 (4 0 0), 4.40	Cubic (*Pm*3*n*)	*a* = 84.44 Å
				*Z* = 104.4
**10**	135	43.42 (2 0 0), 38.65 (2 1 0), 35.25 (2 1 1), 23.88 (3 2 0), 23.05 (3 2 1), 21.58 (4 0 0), 4.48	Cubic (*Pm*3*n*)	*a* = 86.84 Å
				Z = 114.0
**11**	25	36.71 (1 0 0), 21.13 (1 1 0), 18.31 (2 0 0) 4.38	Col_h_	*a* = 42.38 Å
				*S*_h_ = 1555.43 Å^2^
				*V*_h_ = 6812.81Å^3^
				*Z* = 2.4
**14**	50	42.07 (1 0 0), 24.02 (1 1 0), 20.88 (2 0 0), 15.80 (2 1 0), 4.40, 3.44	Col_h_	*a* = 48.57 Å
				*S*_h_ = 2042.99 Å^2^
				*V*_h_ = 7027.89Å^3^
				*Z* = 0.8
**14**	110	37.69 (2 0 0), 23.73 (3 1 0), 16.50 (4 2 0), 4.51	Cubic (*Pm*3*n*)	*a* = 75.38 Å
				*Z* = 50.7
**15**	186	46.96 (2 0 0), 41.75 (2 1 0), 38.49 (2 1 1), 25.56 (3 2 1), 23.53 (4 0 0), 4.50	Cubic (*Pm*3*n*)	*a* = 93.93 Å
				*Z* = 46.85
**16**	28	44.67 (1 0 0), 25.79 (1 1 0), 22.49 (2 0 0), 16.95 (2 1 0), 14.89 (3 0 0), 12.44 (3 1 0), 4.33, 3.57	Col_h_	*a* = 51.58 Å
				*S*_h_ = 2304.05 Å^2^
				*V*_h_ = 8225.48 Å^3^
				*Z* = 0.9
**16**	150	40.56 (2 0 0), 36.36 (2 1 0), 33.18 (2 1 1), 21.65 (3 2 1), 20.22 (4 0 0), 15.09 (5 2 1), 4.59	Cubic (*Pm*3*n*)	*a* = 81.12Å
				Z = 61.4
**17**	170	64.86 (2 0 0), 46.16 (2 2 0), 41.27 (3 1 0), 37.74 (2 2 2), 32.51 (4 0 0), 29.15 (4 2 0), 26.65 (4 2 2), 25.50 (5 0 0), 24.69 (5 1 1), 23.06 (4 4 0), 18.95 (6 3 1), 18.09 (7 1 1), 17.09 (7 2 2), 4.54	Cubic (*Pm*3*n*)	*a* = 129.73Å
				Z = 121.78

a Hexagonal: (lattice area **S**_**h**_ = (√3/2)­a^2^; lattice volume **V**_**h**_ = S_h_ h_c_ (**h**_**a**_ if **h**_**c**_ is not observed); No. of molecules per slice of column (**Z**) = N_a_ **ρV**_**h**_/M; **N**_**a**_ = Avogadro’s number; **ρ** = density; **a**=lattice parameter; **h**_**c**_=core core peak; **h**_**a**_ chain peak; **M** = molecular weight). Cubic (*Pm*3*n*): **a =** lattice parameter; No. of molecules per unit cell in the cubic lattice (Z) = a^3^ρN_A_/M; **ρ** it is assumed to be 1 gcm^–3^.

**Figure 9:**
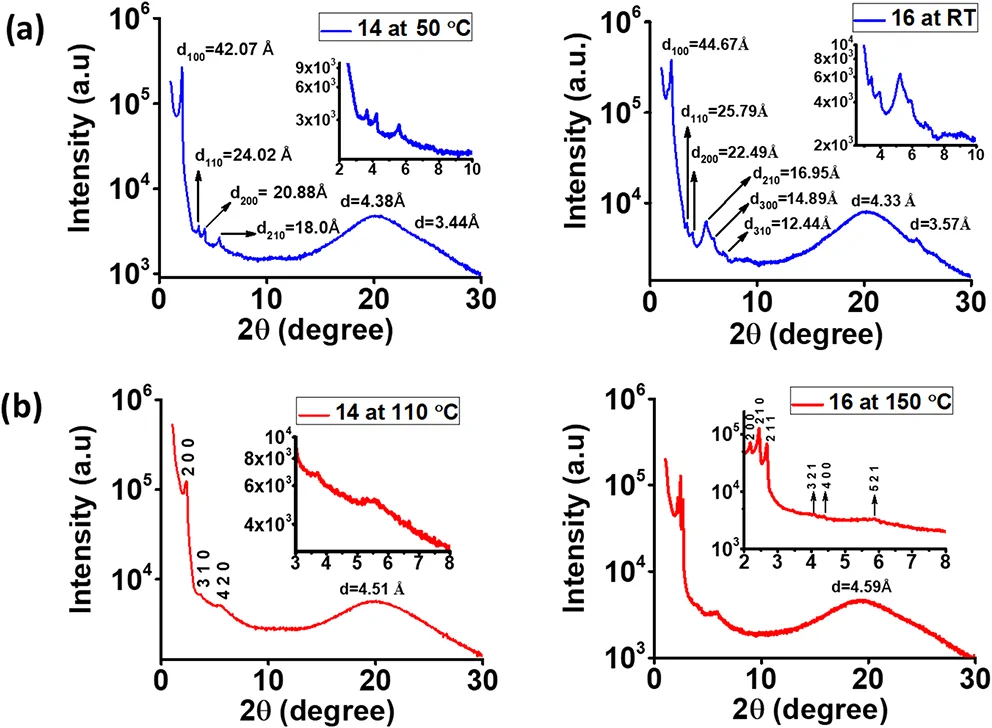
X-ray diffraction patterns of **14** and **16** obtained upon cooling from the isotropic (Iso)­phase, showing the columnar hexagonal (Col_h_) phase at low temperatures (a) and the cubic (*Pm*3*n*) mesophase at higher temperatures (b).

For compounds **15** and **17**, the X-ray diffractograms show that the supramolecular assembly exists in the *Pm*3*n* cubic phase. Multiple sharp peaks are observed in the small-angle region, in the ratios of 2:√5:​√6:√14:​√16 and 2:√8:​√10:√12:​√16:√20:​√24:√25:​√27:√32:​√46:√51:​√57, which can be indexed on the *Pm*3*n* cubic lattice. The lattice parameter (a) of the cubic phase is found to be 93.93 Å and 129.73 Å, respectively, for compounds **15** and **17**. In the wide-angle region, a broad peak is observed at 4.5 Å, corresponding to molten alkyl chains. The number of molecules in the unit cell of the cubic lattice is 47 and 122. The details of the indexing are given in Table 3, and the X-ray diffractograms are presented in Figure 10. X-ray data from the cubic phase of all compounds can be indexed on the *Pm*3*n* space group and are given in Table S2.

**Figure 10:**
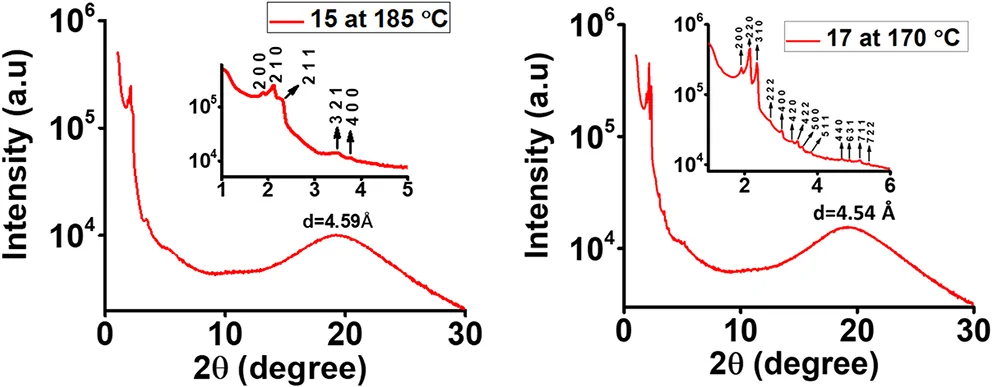
X-ray diffraction patterns of **15** at 185 °C and **17** at 170 °C, in the cubic phase.

## Conclusion

In summary, first and second-generation carboxylic acid–functionalized dendrimers were successfully synthesized via CuAAC click chemistry and assembled with benzotriazole or benzimidazole derivatives through hydrogen bonding to form well-defined supramolecular liquid crystalline systems. Hydrogen bonding has been employed to induce mesomorphism in liquid-crystalline systems, primarily yielding nematic, smectic, or columnar phases. The present work demonstrates, to the best of our knowledge, for the first time that hydrogen-bonded complexes of carboxylic acid–functionalized dendrimers with benzotriazole or benzimidazole derivatives can induce both columnar hexagonal and cubic mesophases within the same supramolecular system, with the mesophase type governed by dendrimer generation. The first-generation dendrimers, containing carboxylic acid and hydroxyl groups, exhibit a columnar hexagonal (Col_h_) mesophase, while the second-generation dendrimers bearing carboxylic acid and ester functionalities display a cubic (*Pm*3*n*) mesophase, as evidenced by POM, DSC, and X-ray diffraction analysis. This generation dependence is retained in the hydrogen-bonded complexes: complexes derived from the first-generation dendrimer (14 and 16) exhibit a columnar hexagonal mesophase at room temperature with a transition to a cubic phase at elevated temperature, whereas complexes derived from the second-generation dendrimer (**15** and **17**) retain only the cubic phase across the entire mesomorphic range, mirroring the behavior of the parent dendrimers. This consistent generation-dependent mesophase switching, in both the dendrimers and their hydrogen-bonded complexes, represents a systematic and tunable approach to controlling liquid-crystalline phase behavior, distinct from previously reported hydrogen-bonded dendritic systems. The formation of stable 1:3 hydrogen-bonded complexes was confirmed by FTIR and ^1^H NMR spectroscopy, and all complexes retain mesomorphic order. Most significantly, complexes **14** and **16** exhibit stable room-temperature mesomorphism, a practical feature not observed in previously reported hydrogen-bonded dendrimer systems. Taken together, these results show that combining controlled dendrimer generation with hydrogen-bond-driven supramolecular assembly offers a route to tunable, room-temperature mesomorphic dendritic liquid crystals, with potential applications in switching devices, optical data storage, and optoelectronics.

## Supplementary Material



## Notes

Experimental procedures, synthetic scheme, ^1^H and ^13^C NMR spectra, IR spectra (PDF)

## §

Department of Industrial Engineering, Swanson School of Engineering, University of Pittsburgh, Pittsburgh, Pennsylvania 15261, United States
