# Supplementary material for: Room-Temperature Columnar Liquid Crystals from Hydrogen-Bonded Dendritic Assemblies
Source: ACS Omega. 2026 Aug 1;11(32):48361–72. doi: 10.1021/acsomega.6c04985 (PMC13491994; doi:10.1021/acsomega.6c04985)
Supplement: Supplementary file 1 [file ao6c04985_si_001.pdf]

Supporting information

Room-Temperature Columnar Liquid Crystals from Hydrogen-Bonded Dendritic Assemblies.

Sivakumar Irla,[1,3*] Velayudhan A. Raghunathan,[1] and Sandeep Kumar[1,2,*]

AUTHOR ADDRESS

1. Raman Research Institute, Soft Condensed Matter, C. V. Raman Avenue, Sadashivanagar, Bangalore – 560080, India.

2. Department of Chemistry and Biochemistry, Sharda School of Engineering and Science, Sharda University, Greater Noida - 201310, India.

*E-mail: skumar.sandeep@gmail.com.

3. Present address: Department of Industrial Engineering, Swanson School of Engineering, University of Pittsburgh, Pittsburgh, Pennsylvania 15261, United States.

*E-mail: iss133@pitt.edu;

Table of contents:

1. Experimental materials and methods 3 – 3
2. Synthetic scheme of acid-functionalized 1st and 2nd generation dendrimers and their hydrogen-bonded complexes (HB-complexes) 4 – 7
3. Synthesis Procedures and characterization 8 – 14
4. FTIR spectra of dendrimers (10, 12, 13) and HB complex (15, 17), and IR stretching values of compounds 10 -17. 15 – 15
5. Comparative ^{1}H NMR studies of **10**, **11, 13** pure compounds, and HB Complex of **16** and **17** in $CDCl_3$ solution. 16 –16
6. ^{1}H and ^{13}C-NMR spectra of all compounds 17 – 33

1. Experimental Materials and Methods

All the chemicals and reagents are AR-grade quality and purchased from Sigma-Aldrich. The solvents were dried and distilled using standard protocols. The intermediate and final compounds were purified by column chromatography using silica gel (100–200) mesh and recrystallization from the suitable solvent. The spectral and elemental analysis confirmed the structure and purity of all compounds. ^{1}H Nuclear magnetic resonance spectroscopy (NMR) and ^{13}C NMR were recorded on Bruker 500 MHz machine using deuterated chloroform ($CDCl_3$) as a solvent, and chemical shifts are recorded in ppm from tetramethylsilane as internal standard ($CDCl_3$: ^{1}H NMR: $\delta = 7.23$; ^{13}C NMR = 77.0). Data were reported as: s = singlet, d = doublet, t = triplet m = multiplet, bd = broad doublet. Elemental analysis was carried out using Elementary Vario MICRO Select instrument. The microscopic textures were recorded on a sample placed between ordinary glass slides using an Olympus BX51 polarizing optical microscope (Olympus, Tokyo, Japan) in conjunction with Mettler FP82HT hot stage and Mettler FP90 central processor. The liquid crystalline samples' phase transition temperature and associated enthalpies were determined by the Mettler Toledo DSC instrument using 2–4 mg samples and a scan rate of 10°C min^{-1}. Further detailed examination of liquid crystalline mesophase structure is done by X-ray diffraction using Panalytical (Empyrean) Cu-Kα (1.54 Å) X-ray diffractometer. Thermal stability was confirmed by the Perkin Elmer TGA4000 analyzer.

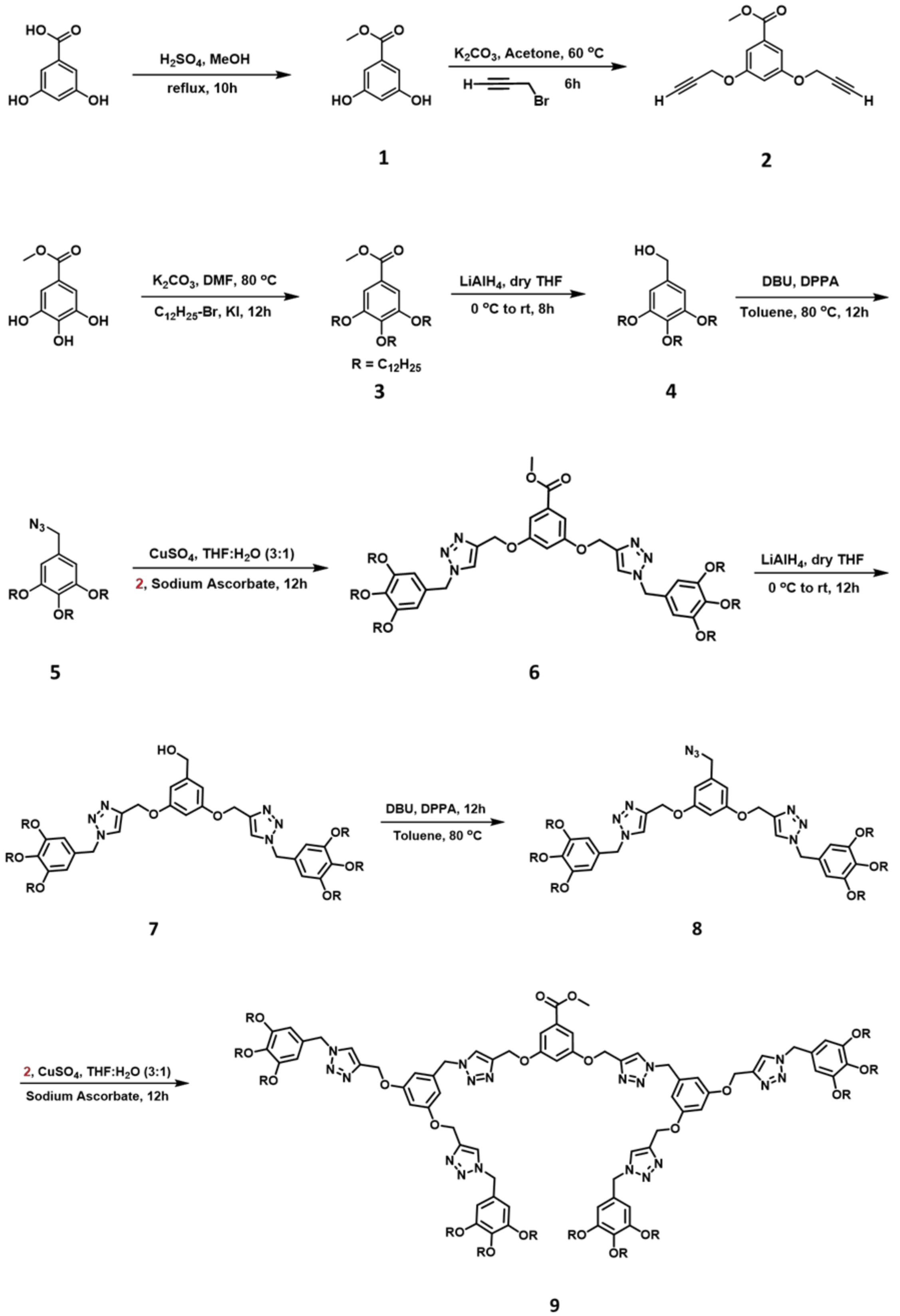

HO O
HO OH
H2SO4, MeOH
reflux, 10h
HO OH
1
K2CO3, Acetone, 60 oC
H Br
6h
H O O H
2
HO OH
OH
K2CO3, DMF, 80 oC
C12H25-Br, KI, 12h
RO OR
OR
R = C12H25
3
LiAlH4, dry THF
0 oC to rt, 8h
HO
RO OR
OR
4
DBU, DPPA
Toluene, 80 oC, 12h
N3
RO OR
OR
5
CuSO4, THF:H2O (3:1)
2, Sodium Ascorbate, 12h
6
LiAlH4, dry THF
0 oC to rt, 12h
HO
7
DBU, DPPA, 12h
Toluene, 80 oC
N3
8
2, CuSO4, THF:H2O (3:1)
Sodium Ascorbate, 12h
9

9

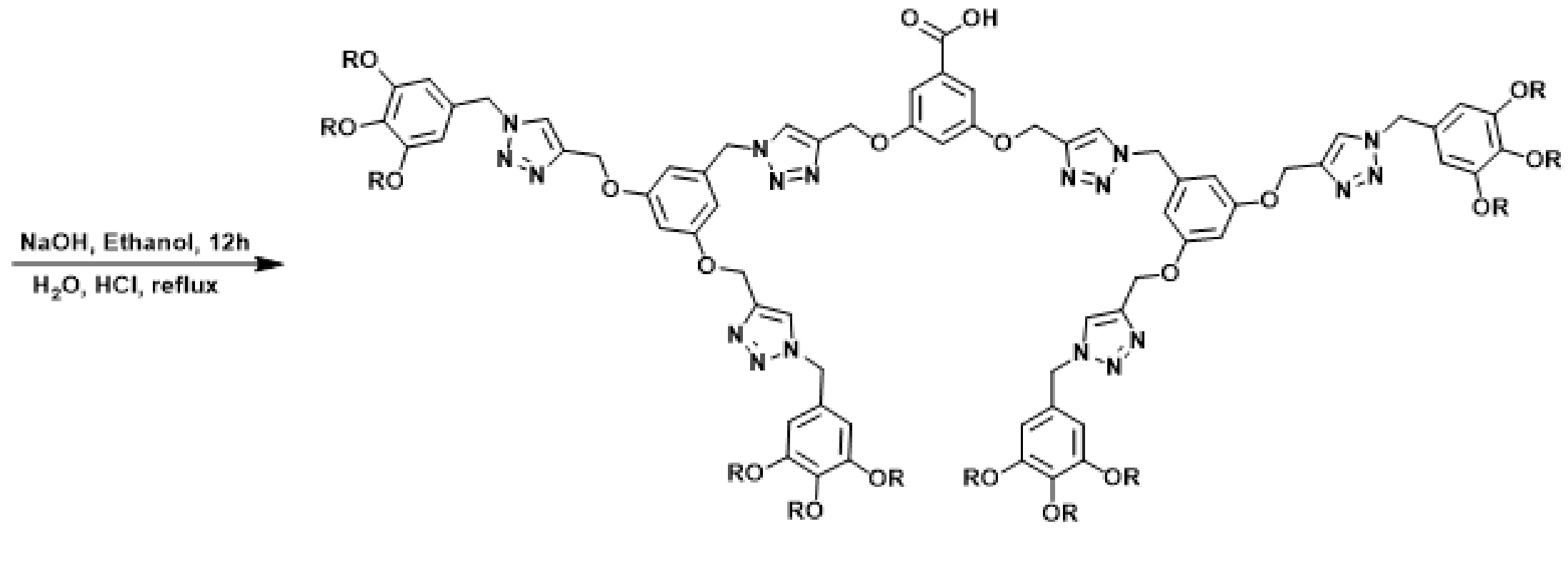
NaOH, Ethanol, 12h
H_2O, HCl, reflux

10

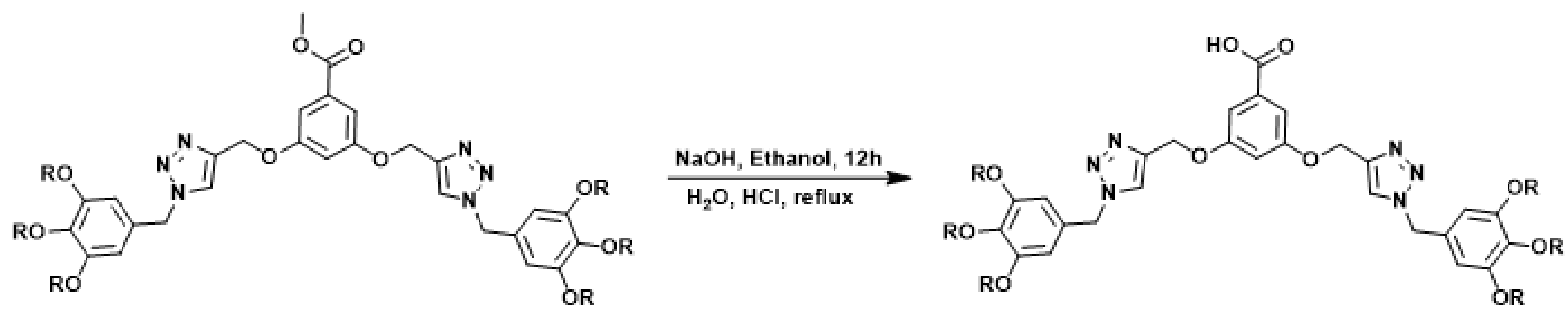
NaOH, Ethanol, 12h
H_2O, HCl, reflux

6

11

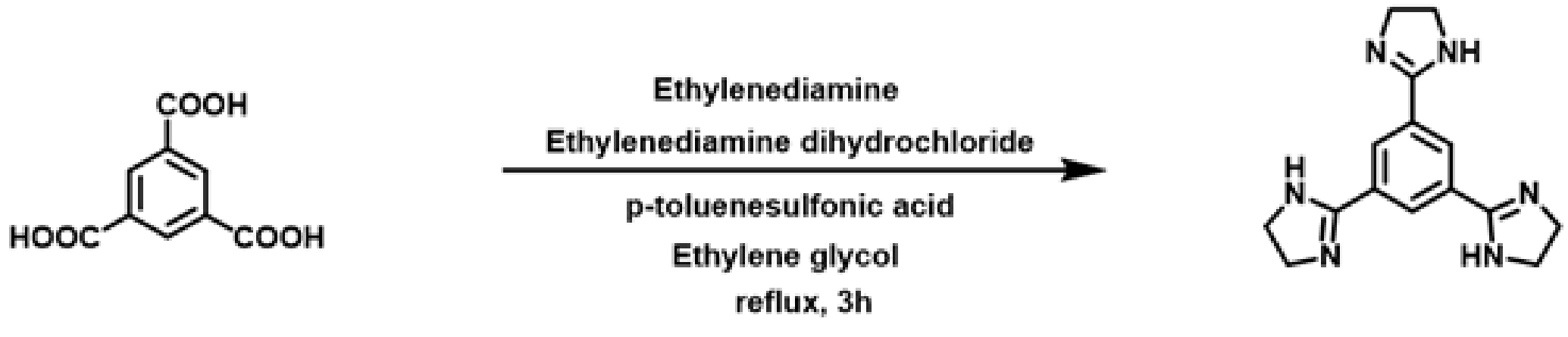
COOH
HOOC
COOH
Ethylenediamine
Ethylenediamine dihydrochloride
p-toluenesulfonic acid
Ethylene glycol
reflux, 3h

12

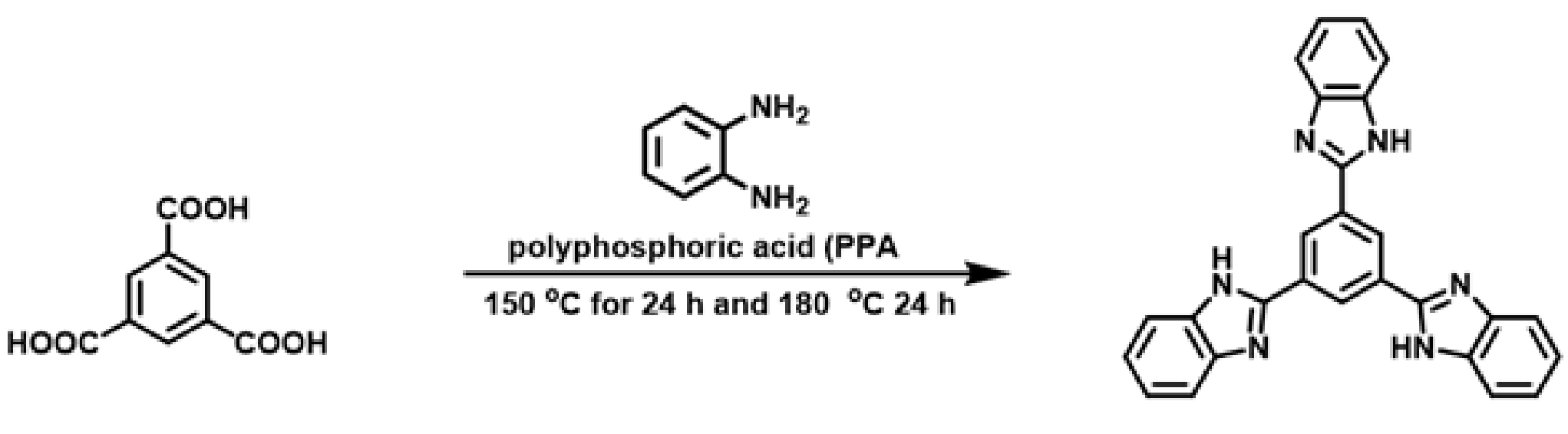
COOH
HOOC
COOH
NH_2
NH_2
polyphosphoric acid (PPA
150 °C for 24 h and 180 °C 24 h

13

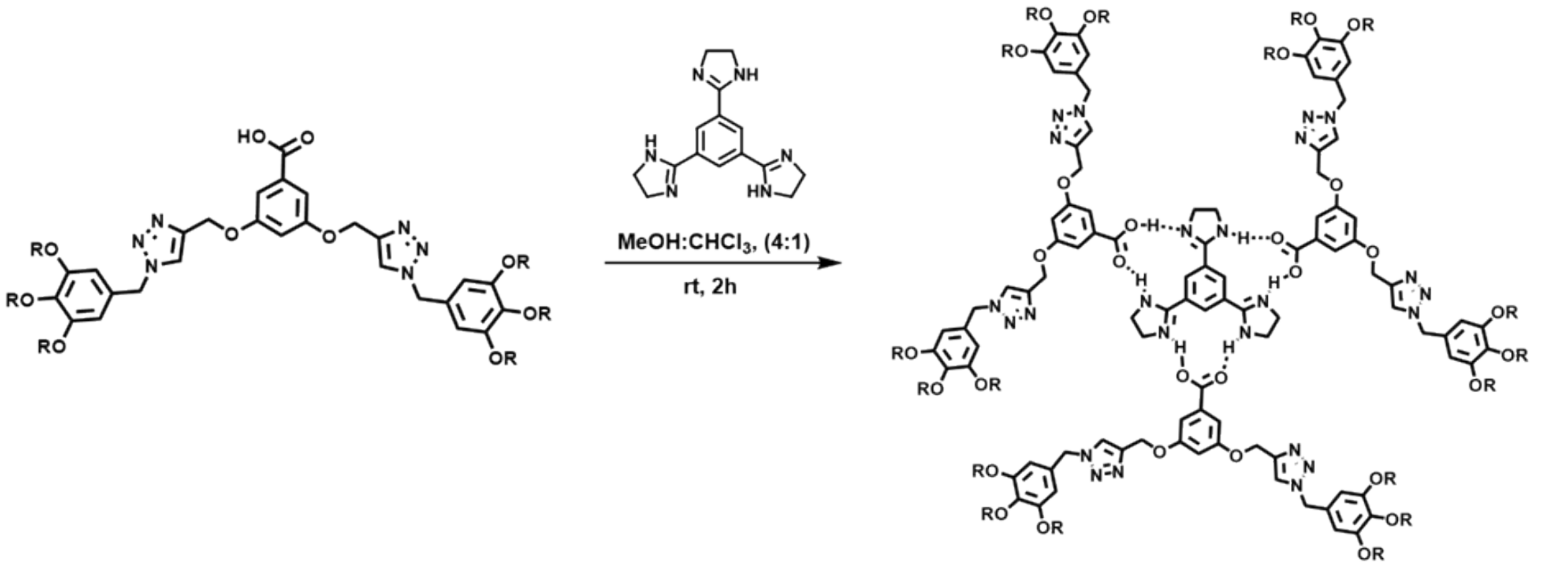

14

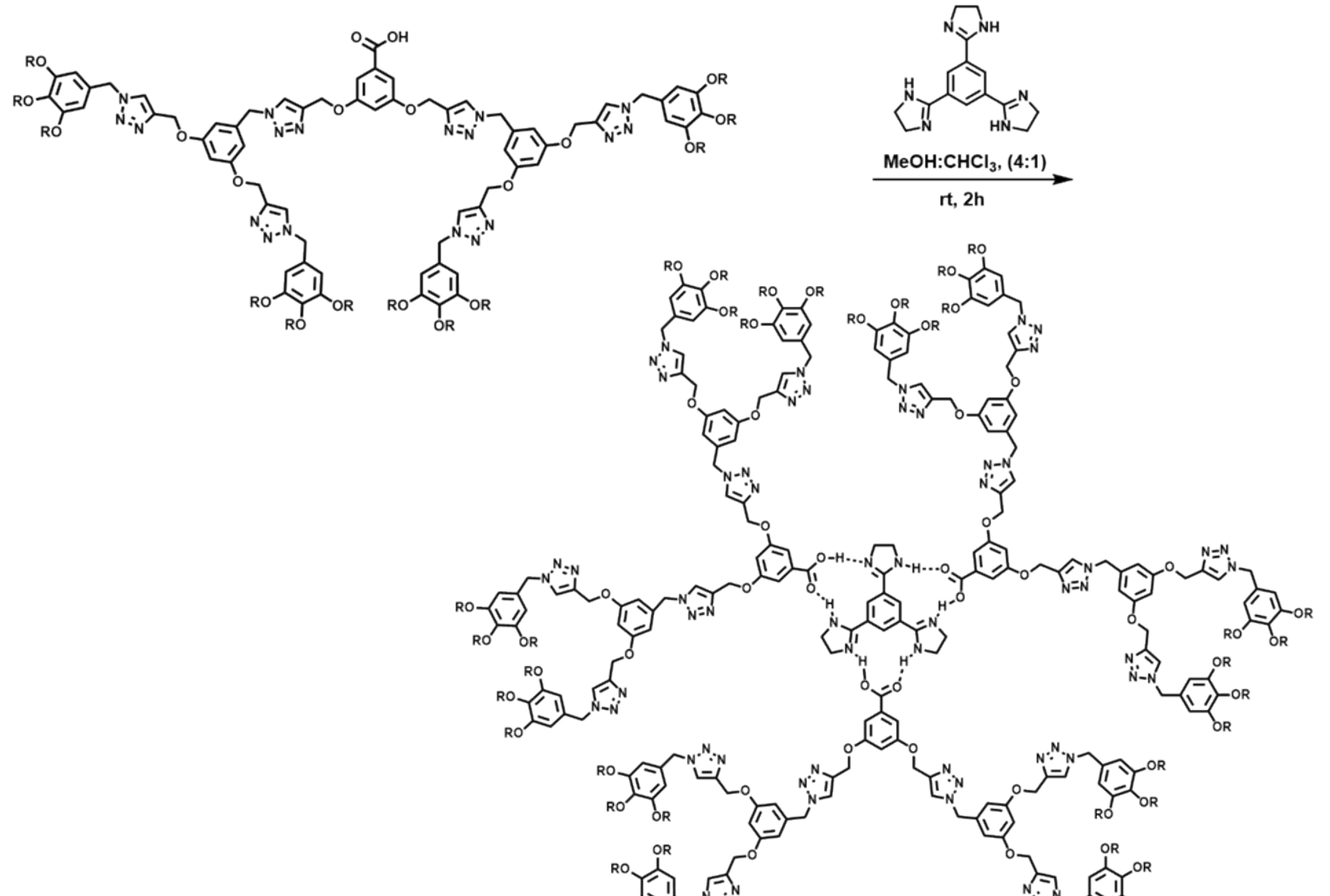

15

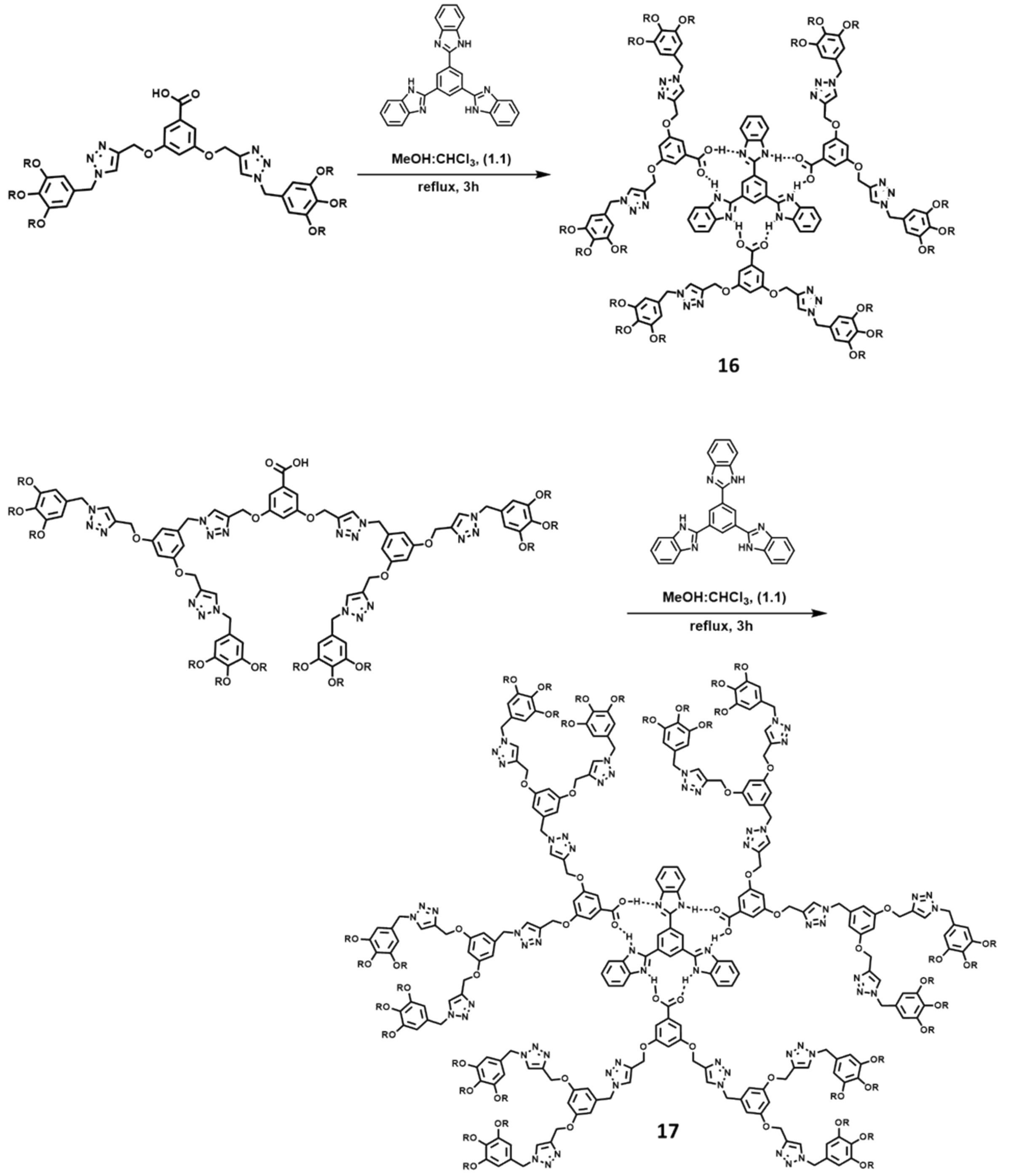

Scheme S1 . Synthetic scheme of acid-functionalized 1st and 2nd generation dendrimers and their hydrogen-bonded complexes.

3. Synthesis Procedure and characterization:

Compound 1: To a solution of 3,5-dihydroxybenzoic acid (10.0 g, 64.88 mmol, 1 eq) in methanol (200 mL) was added concentrated sulfuric acid (13.8 mL, 25.45 g, 129.76 mmol, 2 eq). The solution was allowed to reflux for 8 h. Completion of the reaction was confirmed by TLC, and the solvent was removed using a rotavapor. After that, the crude solid was dissolved in ethyl acetate, washed with $NaHCO_3$ solution, dried over anhydrous Na_2SO_4, and the solvent was evaporated to give the desired product. Yield: 92%; ^{1}H NMR (500 MHz, Methanol-d$_4$): δ (ppm) = 6.94 (d, J = 2.50 Hz, 2H), 6.50 (t, J = 4.50 Hz, 1H), 3.86 (s, 3H); ^{13}C NMR (125 MHz, $CDCl_3$): δ ppm = 168.93, 159.70, 133.06, 109.00, 108.45, 52.77. Elemental analysis: C, 57.14; H, 4.80 calculated (%): C, 57.64; H, 5.10(expt. %).

Compound 2: To a solution of methyl 3,5-dihydroxybenzoate (8 g, 47.57 mmol, 1 eq) in acetone (150 mL), K_2CO_3 (19.72 g, 142.72 mmol, 3 eq) was added. Then the reaction mixture was stirred for 30 min at room temperature under nitrogen. Propargyl bromide (80% in toluene, 20.47 mL) was added, and the reaction mixture was kept at 80 ºC for 6 h. After completion of the reaction, the solvent was removed under reduced pressure, and the residue was extracted with $CHCl_3$. The organic layer was dried over anhydrous Na_2SO_4. The pure product was obtained by column chromatography using petroleum ether and dichloromethane (8:2) as the eluent, and dried under high vacuum to obtain the desired product as a white solid. Yield: 92%; ^{1}H NMR (500 MHz, $CDCl_3$): δ (ppm) = 7.29 (d, J = 1.0 Hz, 2H), 6.81 (s, 1H), 4.71 (d, 4H), 3.91 (s, 3H), 2.54 (s, 2H); ^{13}C NMR (125 MHz, $CDCl_3$): δ ppm = 166.46, 158.50, 132.15, 108.88, 107.51, 77.97, 76.00, 56.12, 52.37. Elemental analysis: C, 68.85; H, 4.95 calculated (%): C, 68.63; H, 4.93(expt. %).

Compound 3: In a round bottom flask, ethyl gallate (10 g, 198.17 mmol, 1 eq) was dissolved in 500 mL of DMF, K_2CO_3 (41.84 g, 302.76 mmol, 6 eq) was added to it and stirred for 15 minutes, 1-Bromododecane (50.30 g, 201.84 mmol, 4 eq) and Potassium iodide (catalytic amount) were added to it the reaction mixture was refluxed for overnight. After completion of the reaction, the mixture was cooled to room temperature, then poured into ice water, and the precipitate was filtered. The crude product was purified by column chromatography using petroleum ether: dichloromethane (8:2) as the eluent and dried under high vacuum to give the desired product as a white solid. Yield: 83%; ^{1}H NMR (500 MHz, $CDCl_3$): δ (ppm) = 7.17 (s, 2H), 3.94 – 3.92 (m, 6H), 3.81 (s, 3H), 1.76 – 1.63 (m, 6H), 1.42 – 1.36 (m, 6H), 1.27 – 1.19 (m, 48H), 0.80 (t, J = 6.25 Hz, 9H); ^{13}C NMR (125 MHz, $CDCl_3$): δ ppm = 166.93, 152.81,

142.33, 124.65, 107.94, 73.47, 69.14, 52.08, 31.94, 30.34, 29.77, 29.74, 29.72, 29.68, 29.65, 29.58, 29.41, 29.39, 29.31, 26.09, 26.07, 22.71, 14.11. Elemental analysis: C, 76.69; H, 11.70 calculated (%): C, 76.85; H, 12.15(expt. %).

Compound 4: In a round bottom flask, compound 3 (5 g, 5.83 mmol, 1 eq), was dissolved in 100 mL of dry tetrahydrofuran and cooled to 0 ºC and $LiAlH_4$ (0.331 g, 8.74 mmol, 1.5 eq), was added to the reaction mixture portion-wise and allowed to room temperature and the reaction mixture kept for overnight, after completion of the reaction, the reaction mixture was quenched. The reaction mixture was extracted with dichloromethane, dried over anhydrous Na_2SO_4, and the solvent was evaporated. The crude product was purified by column chromatography using petroleum ether and dichloromethane (7:3) as the eluent, and dried under high vacuum to give the desired product. Yield: 97%; ^{1}H NMR (500 MHz, $CDCl_3$): δ (ppm) = 6.57 (s, 2H), 4.61 (s, 2H), 4.00 – 3.94 (m, 6H), 1.82 – 1.74 (m, 6H), 1.48 (bm, 6H), 1.28 (bm, 48H), 0.9 (t, *J* = 6.25 Hz, 9H); ^{13}C NMR (125 MHz, $CDCl_3$): δ ppm = 153.25, 137.57, 136.12, 105.36, 73.45, 69.12, 65.59, 31.95, 31.93, 30.33, 29.76, 29.75, 29.71, 29.67, 29.63, 29.44, 29.40, 29.37, 26.15, 26.12, 22.69, 14.10. Elemental analysis: C, 78.12; H, 12.20 calculated (%): C, 78.82.63; H, 12.56(expt. %).

Compound 5: To a solution of 4 (0.5 g, 0.75 mmol, 1 eq) in toluene (10 mL) 1, 8-diazabicyclo (5.4.0) undec-7-ene (DBU) (0.29 g, 1.96 mmol, 2.6 eq) and diphenyl phosphoryl azide (DPPA) (0.44 g, 1.73 mmol, 2.3 eq) were added. Then the reaction mixture was stirred at 80 ºC for 12 h. After completion of the reaction, the reaction mixture was quenched with saturated NH_4Cl solution and extracted with $CHCl_3$. The organic layer was dried over anhydrous Na_2SO_4, and the solvent was removed. The crude product was purified by column chromatography using petroleum ether and dichloromethane (7:3) as the eluent, and dried under high vacuum to give the desired product as a white solid. Yield: 80%; ^{1}H NMR (500 MHz, $CDCl_3$): δ (ppm) = 6.41 (s, 2H), 4.16 (s, 2H), 3.91 – 3.85 (m, 6H), 1.75 – 1.63 (m, 6H), 1.42 – 1.36 (m, 6H), 1.27 – 1.19 (m, 48H), 0.81 (t, *J* = 7.0 Hz, 9H); ^{13}C NMR (125 MHz, $CDCl_3$): δ ppm = 153.36, 138.14, 130.37, 106.61, 73.43, 69.16, 55.19, 31.95, 30.35, 29.78, 29.76, 29.72, 29.69, 29.66, 29.63, 29.44, 29.42, 29.41, 29.39, 26.14, 26.11, 22.71, 14.12. Elemental analysis: C, 75.27; H, 11.61; N, 6.12 calculated (%): C, 75.18; H, 11.93, N, 6.05 (expt. %).

Compound 6: To a solution of 2 (0.9 g, 3.68 mmol, 1 eq) and compound 5 (5.30 g, 7.73 mmol, 2.1 eq) in 150 mL of tetrahydrofuran, water (3:1 ratio) was added. Then the reaction mixture

was stirred for 15min. $CuSO_4$ (0.0294 g, 0.1842 mmol, 0.05 eq) was dissolved in the minimum amount of water and added to the reaction mixture after 5 min. Sodium ascorbate (0.0671 g, 0.339 mmol, 0.092 eq) was dissolved with the minimum amount of water added to the reaction mixture, and the reaction mixture was kept at room temperature for 12 h. After completion of the reaction, the reaction mixture was kept at 0 ºC, diluted with water, 26 mL of ammonia solution was added, stirred for 20 min, and extracted with $CHCl_3$. The organic layer was dried over anhydrous Na_2SO_4. The crude product was purified by column chromatography using petroleum ether and ethyl acetate (9:1) as eluent and dried under a high vacuum to give the desired product as a white solid. Yield: 92%; ^{1}H NMR (500 MHz, $CDCl_3$): δ (ppm) = 7.48 (s, 2H), 7.20 – 7.19 (m, 2H), 6.73 – 6.72 (m, 1H), 6.39 (s, 4H), 5.33 (s, 4H), 5.10 (s, 4H), 3.86 – 3.81 (m, 15H), 1.72 – 1.62 (m, 12H), 1.40 – 1.34 (m, 12H), 1.22 – 1.18 (m, 96H), 0.80 (t, J = 7.0 Hz, 18H),; ^{13}C NMR (125 MHz, $CDCl_3$): δ ppm = 166.49, 159.25, 153.62, 143.86, 138.53, 132.17, 129.20, 122.68, 108.66, 106.73, 73.47, 69.24, 62.26, 54.59, 52.29, 31.95, 31.93, 30.34, 29.76, 29.75, 29.72, 29.67, 29.65, 29.61, 29.44, 29.40, 29.38, 26.12, 26.10, 22.70, 14.11. Elemental analysis: C, 74.30; H, 10.60; N, 5.20 calculated (%): C, 74.36; H, 11.01; N, 5.15 (expt. %).

Compound 7: In a round bottom flask, compound 6 (3 g, 1.88 mmol, 1 eq), was dissolved in 100 mL of dry tetrahydrofuran and cooled to 0 ºC and $LiAlH_4$ (0.107 g, 2.833 mmol, 1.5 eq), was added to the reaction mixture portion wise and allowed to room temperature and the reaction mixture kept overnight, after completion of the reaction, the reaction mixture was quenched methanol. The reaction mixture was extracted with dichloromethane, the organic layer was dried over anhydrous Na_2SO_4, and the solvent was removed by rotavapor. The crude product was purified by column chromatography using petroleum ether and ethyl acetate (7:3) as eluent and dried under a high vacuum to give the desired product as a white solid. Yield: 95%; ^{1}H NMR (500 MHz, $CDCl_3$): δ (ppm) = 7.57 (s, 2H), 6.62 (s, 2H), 6.53 (s, 1H), 6.48 (s, 4H), 5.42 (s, 4H), 5.16 (s, 4H), 4.62 (s, 2H), 3.95 – 3.91 (m, 12H), 1.85 – 1.73 (m, 12H), 1.49 – 1.43 (m, 12H), 1.31 – 1.27 (m, 96H), 0.88 (t, J = 6.75 Hz, 18H); ^{13}C NMR (125 MHz, $CDCl_3$): δ ppm = 159.47, 153.60, 144.21, 143.91, 138.50, 129.23, 122.72, 106.76, 105.86, 100.94, 73.49, 69.25, 64.89, 61.97, 54.59, 31.94, 30.34, 29.77, 29.75, 29.72, 29.71, 29.68, 29.66, 29.62, 29.45, 29.41, 29.38, 26.13, 26.11, 22.71, 14.13. Elemental analysis: C, 74.86; H, 10.79; N, 5.29 calculated (%): C, 74.74; H, 11.32; N, 5.31(expt. %).

Compound 8: To a solution of 7 (7 g, 4.406 mmol, 1 eq) in toluene (100 mL), 1,8-diazabicyclo (5.4.0) undec-7-ene (DBU) (1.801 g, 11.45 mmol, 2.6 eq) and diphenyl phosphoryl azide

(DPPA) (2.60 g, 1.915 mmol, 2.3 eq) were added. Then the reaction mixture was stirred at 80 ºC for 12 h. After completion of the reaction, the reaction mixture was quenched with saturated NH_4Cl solution and extracted with $CHCl_3$. The organic layer was dried over anhydrous Na_2SO_4, and the solvent was removed. The crude product was purified by column chromatography using petroleum ether and ethyl acetate (8:2) as eluent and dried under a high vacuum to give the desired product as a white solid. Yield: 80%; ^{1}H NMR (500 MHz, $CDCl_3$): δ (ppm) = 7.57 (s, 2H), 6.60 (s, 1H), 6.56 (s, 2H), 6.48 (s, 4H), 5.42 (s, 4H), 5.17 (s, 4H), 4.26 (s, 2H), 3.95 – 3.91 (m, 12H), 1.82 – 1.67 (m, 12H), 1.49 – 1.43 (m, 12H), 1.31 – 1.27 (m, 96H), 0.89 (t, J = 6.5 Hz, 18H),; ^{13}C NMR (125 MHz, $CDCl_3$): δ ppm = 159.66, 153.62, 144.03, 138.52, 137.79, 129.19, 122.69, 107.49, 106.75, 101.53, 73.48, 69.24, 62.12, 54.65, 54.62, 31.94, 30.33, 29.77, 29.75, 29.72, 29.67, 29.65, 29.62, 29.44, 29.41, 29.38, 26.12, 26.10, 22.71, 14.13. Elemental analysis: C, 73.70; H, 10.56; N, 7.81 calculated (%): C, 73.75; H, 10.48; N, 7.74 (expt. %).

Compound 9: To a mixture of 2 (0.5 g, 2.047 mmol, 1 eq) and compound 8 (6.93 g, 4.29 mmol, 2.1 eq) in 100 mL of tetrahydrofuran: water (3:1 ratio), was added. Then the reaction mixture was stirred for 15 min. $CuSO_4$ (0.0163 g, 0.102 mmol, 0.05 eq) was dissolved with the minimum amount of water and added to the reaction mixture after 5 min. Sodium ascorbate (0.0373 g, 0.188 mmol, 0.092 eq) was dissolved in the minimum amount of water, added to the reaction mixture, and then kept at room temperature for 12 h. After completion of the reaction, the reaction mixture was kept at 0 ºC, diluted with water, 26 mL of ammonia solution was added, stirred for 20 min, and extracted with $CHCl_3$. The organic layer was dried over anhydrous Na_2SO_4. The crude product was purified by column chromatography using petroleum ether and ethyl acetate (9:1) as the eluent, and dried under high vacuum to obtain the desired product as a white solid. Yield: 92%; ^{1}H NMR (500 MHz, $CDCl_3$): δ (ppm) = 7.59 (s, 2H), 7.55 (s, 4H), 7.26 (s, 2H), 6.80 (s, 1H), 6.59 (s, 2H), 6.48 – 6.47 (m, 12H), 5.42 – 5.39 (m, 12H), 5.16 (s, 4H), 5.08 (s, 8H), 3.93 – 3.88 (m, 24H), 3.86 (s, 3H), 1.79 – 1.69 (m, 24H), 1.61(bs, 24H) 1.46 – 1.41 (m, 24H), 1.29 – 1.25 (m, 168H), 0.87 (t, J = 6.5 Hz, 36H), ^{13}C NMR (125 MHz, $CDCl_3$): δ ppm = 166.49, 159.86, 159.21, 153.61, 143.95, 143.72, 138.52, 136.81, 132.16, 129.26, 123.07, 122.89, 108.76, 107.42, 106.91, 106.77, 101.91, 73.48, 69.26, 62.19, 61.99, 54.57, 54.09, 52.27, 31.95, 31.94, 30.35, 29.77, 29.72, 29.67, 29.66, 29.63, 29.45, 29.38, 26.12, 22.70, 14.12. . Elemental analysis: C, 73.36; H, 10.16; N, 7.26 calculated (%): C, 73.02; H, 9.66; N, 7.25 (expt. %).

General Procedure for the synthesis of 10 and 11

In a round-bottom flask, 9 or 10 (1 eq) was dissolved in ethanol, NaOH (1.3 eq) was added, and the mixture was refluxed overnight. After completion of the reaction, the reaction mixture was acidified (pH = 1-2) with concentrated HCl. The precipitate was observed, filtered using a Buchner funnel, and the residue was dissolved in $CHCl_3$ and washed with a brine solution, followed by water. The reaction mixture was extracted with $CHCl_3$; the organic layer was dried over anhydrous Na_2SO_4. The solvent was evaporated, and the crude product was purified by column chromatography using petroleum ether and ethyl acetate as eluent (8:2 v/v), then dried under high vacuum to obtain the desired product.

Compound 10: Compound 9 (4 g, 1.152 mmol, 1 eq) and NaOH (0.059 g, 1.498 mmol, 1.3 eq) in 100 mL of ethanol Yield: 90%; ^{1}H NMR (500 MHz, $CDCl_3$): δ (ppm) = 7.55 (s, 4H), 7.50 (s, 2H), 7.19 (s, 2H), 6.78 (s, 1H), 6.56 (s, 2H), 6.48 – 6.45 (m, 12H), 5.39 (s, 12H), 5.22 (s, 4H), 5.07 (s, 8H), 3.93 – 3.89 (m, 24H), 1.79 – 1.69 (m, 24H), 1.47 – 1.42 (m, 24H), 1.25 (m, 192H), 0.87 (t, J = 6.5 Hz, 36H),; ^{13}C NMR (125 MHz, $CDCl_3$): δ ppm = 159.86, 158.95, 153.62, 144.05, 143.62, 138.53, 136.80, 129.12, 123.02, 122..89, 108.93, 108.26, 107.40, 106.82, 101.81, 73.51, 69. 27, 62.34, 61.80, 54.67, 54.05, 31.95, 31.93, 30.34, 29.77, 29.72, 29.67, 29.66, 29.62, 29.45, 29.40, 29.38, 26.12, 26.11, 22.70, 14.13. Elemental analysis: C, 73.31; H, 10.15; N, 7.29 calculated (%): C, 72.79; H, 10.07, N, 7.07 (expt. %).

Compound 11: Compound 6 (1 g, 0.618 mmol, 1 eq) and NaOH (0.032 g, 0.804 mmol, 1.3 eq) in 100 mL of ethanol Yield: 92%; ^{1}H NMR (500 MHz, $CDCl_3$): δ (ppm) = 7.49 (s, 2H), 7.26 (s, 2H), 6.76 (bs, 1H), 6.40 (s, 4H), 5.34 (s, 4H), 5.11 (s, 4H), 3.86 – 3.82 (m, 12H), 1.72 – 1.62 (m, 12H), 1.40 – 1.34 (m, 12H), 1.24 – 1.18 (m, 96H), 0.80 (t, J = 7.0 Hz, 18H),; ^{13}C NMR (125 MHz, $CDCl_3$): δ ppm = 170.08, 159.23, 153.62, 143.82, 138.52, 131.86, 129.17, 122.79, 109.13, 107.39, 106.74, 73.48, 69.24, 62.17, 54.64, 31.95, 31.93, 30.34, 29.77, 29.76, 29.72, 29.67, 29.65, 29.62, 29.44, 29.41, 29.38, 26.13, 26.11, 22.70, 14.12. Elemental analysis: C, 74.20; H, 10.57; N, 5.24 calculated (%): C, 74.02; H, 10.79; N, 5.22 (expt. %).

Compound 12: Benzene-1, 3,5-tricarboxylic acid (2 g, 9.15 mmol, 1 eq), ethylenediamine (2.0 g, 33.30 mmol, 3.5 eq), ethylenediamine dihydrochloride (4.42 g, 33.30 mmol, 3.5 eq), toluene-*p*-sulfonic acid (0.127 g, 0.742 mmol, 0.078 eq) and ethylene glycol (14 mL) were added and reflux for 3 h. After three hours, about half of the ethylene glycol was then slowly removed by distillation. The reaction mixture residue was dissolved in water (52 mL) and conc. HCl (4mL). An addition of 50% aqueous NaOH was added until a yellow precipitate was observed.

Reprecipitation was performed using methanol, and the product was dried under high vacuum to obtain the desired product. Yield: 40% ^{1}H NMR (500 MHz, Methanol-d_4): δ (ppm) = 8.24 (s, 3H), 3.80 (s, 12H); ^{13}C NMR (125 MHz, Methanol-d_{43}): δ ppm = 165.01, 130.91, 127.88, 49.30. Elemental analysis: C, 63.81; H, 6.43; N, 29.77 calculated (%): C, 62.91; H, 6.46; N, 29.43 (expt. %).

Compound 13: Benzene-1, 3, 5-tricarboxylic acid (2 g, 9.15 mmol, 1 eq), o-phenyl diamine (3.18 g 29.49 mmol, 3.1 eq), Phosphoric acid (H_3PO_4) 30 mL was added, the reaction mixture kept at 150 ºC for 24 h then the temperature was increased till 180 ºC kept for 18 h, after the pH of the resulting mixture was adjusted to 8 – 9 with NaOH solution, then a large amount of solid precipitated. After filtration, the crude product was obtained and then further purified by recrystallization with hot methanol to give the desired product. ^{1}H NMR (500 MHz, DMSO-d_6): δ (ppm) = 13.39 (s, 3H), 9.11 (s, 3H), 7.76 (d, J = 7.50 Hz, 3H), 7.61 (d, J = 7.50 Hz, 3H), 7.28 (p, J = 7.25 Hz, 6H); ^{13}C NMR (125 MHz, Methanol-d_{43}): δ ppm = 150.79, 144.23, 135.78, 132.27, 125.91, 123.45, 122.49, 119.41, 112.22. Elemental analysis: C, 76.04; H, 4.25; N, 19.71 calculated (%): C, 76.41; H, 4.50; N, 18.03 (expt. %).

Hydrogen-bonding complex of 14 and 15: The 1:3 LC complexes were formed by first dissolving compounds 12 and 10 or 11 in a MeOH / $CHCl_3$ mixture (1:4 v/v), stirred for 2 h, followed by solvent removal using rotavapor dried under high vacuum to get the desired complex.

Complex 14: Compound 12 (0.010 g, 0.0354 mmol, 1 eq), compound 11 (0.170 g, 0.1062 mmol, 3 eq) MeOH / $CHCl_3$ mixture (1:4 v/v) 5 mL; ^{1}H NMR (500 MHz, $CDCl_3$): δ (ppm) = 9.99 (s, 3H), 7.55 (s, 6H), 7.36 (s, 6H), 6.67 (s, 3H), 6.46 (s, 12H), 5.39 (s, 12H), 5.18 (s, 12H), 4.15 (s, 12H), 3.93 – 3.89 (m, 36H), 1.79 – 1.69 (m, 36H), 1.45 – 1.44 (m, 36H), 1.25 (m, 294H), 0.8 (t, J = 6.25 Hz, 54H); ^{13}C NMR (125 MHz, $CDCl_3$): δ ppm = 172.83, 163.10, 158.98, 153.61, 144.37, 139.85, 138.51, 134.73, 129.24, 125.49, 122.65, 108.45, 106.71, 104.95, 73.48, 69.25, 62.18, 54.59, 45.57, 31.94, 31.93, 30.34, 29.76, 29.71, 29.67, 29.65, 29.63, 29.44, 29.40, 29.38, 26.13, 26.11, 22.70, 14.13. Elemental analysis: C, 73.63; H, 10.34; N, 6.60 calculated (%): C, 72.83; H, 10.55; N, 6.57 (expt. %).

Complex 15: Compound 12 (0.010 g, 0.0354 mmol, 1 eq), compound 10 (0.367 g, 0.1062 mmol, 3 eq) MeOH / $CHCl_3$ mixture (1:4 v/v) 5 mL; ^{1}H NMR (500 MHz, $CDCl_3$): δ (ppm) = 9.63 (s, 3H), 7.60 (s, 6H), 7.56 (s, 12H), 7.34 (s, 6H), 6.66 (s, 3H), 6.58 (s, 6H), 6.46 (s, 36H),

5.39 – 5.37 (m, 36H), 5.17 (s, 12H), 5.06 (s, 24H), 4.06 (bs, 12H), 3.92 – 3.87 (m, 72H), 1.78 – 1.68 (m, 72H), 1.46 – 1.40 (m, 72H), 1.25 (m, 582H), 0.86 (t, J = 6.25 Hz, 108H); ^{13}C NMR (125 MHz, $CDCl_3$): δ ppm = 171.68, 162.19, 158.80, 157.89, 152.55, 143.37, 142.66, 138.88, 137.40, 135.90, 128.30, 124.98, 122.13, 121.94, 107.52, 106.33, 105.68, 103.98, 100.78, 72.45, 68.19, 60.98, 60.92, 53.51, 52.98, 45.11, 30.91, 29.33, 28.75, 28.70, 28.65, 28.64, 28.61, 28.43, 28.38, 28.36, 25.10, 21.68, 13.10. Elemental analysis: C, 73.05; H, 10.05; N, 7.89 calculated (%): C, 72.89; H, 9.93; N, 7.73 (expt. %).

Hydrogen-bonding complex of 16 and 17: The 1:3 LC complexes were formed by first dissolving the compound 13 and compound 10 or 11 in a MeOH / $CHCl_3$ mixture (1:4 v/v), stirred for 4 h at 60 ºC followed by solvent removal using rotavapor dried under high vacuum to get the desired complex.

Complex 16: Compound 13 (0.010 g, 0.0234 mmol, 1 eq), compound 11 (0.112 g, 0.070 mmol, 3 eq) MeOH / $CHCl_3$ mixture (1:4 v/v) 5 mL; ^{1}H NMR (500 MHz, $CDCl_3$ + DMSO-d6): δ (ppm) = 9.14 (s, 3H), 8.02 – 7.97 (m, 9H), 7.66 (s, 6H), 7.25 – 7.20 (m, 6H), 6.87 (bs, 3H), 6.54 – 6.53 (m, 15H), 5.44 (s, 12H), 5.15 (s, 12H), 3.88 – 3.84 (m, 36H), 1.74 – 1.72 (m, 24H), 1.68 – 1.65 (m, 12H), 1.44 (m, 36H), 1.25 (m, 294H), 0.86 – 0.85 (m, 54H); ^{13}C NMR (125 MHz, $CDCl_3$ + DMSO-d6): δ ppm = 172.17, 164.06, 158.00, 155.66, 147.97, 142.52, 138.08, 136.87, 135.20, 128.95, 128.82, 113.45, 111.37, 111.04, 77.81, 73.59, 66.64, 58.71, 36.58, 35.06, 34.37, 34.32, 34.30, 34.10, 34.02, 30.85, 27.35, 18.99. Elemental analysis: C, 74.35; H, 10.05; N, 6.42 calculated (%): C, 74.27; H, 10.32; N, 6.10 (expt. %).

Complex 17: Compound 13 (0.010 g, 0.0234 mmol, 1 eq), compound 10 (0.243 g, 0.070 mmol, 3 eq) MeOH / $CHCl_3$ mixture (1:4 v/v) 5 mL. ^{1}H NMR (500 MHz, $CDCl_3$ + DMSO-d6): δ (ppm) = 9.15 (s, 3H), 7.92 – 7.90 (m, 6H), 7.86 – 7.84 (m, 15H), 7.78 – 7.74 (m, 3H), 7.66 (bs, 5H), 7.25 – 7.24 (m, 5H), 6.83 (s, 3H), 6.60 (s, 7H), 6.52 (m, 40H), 5.45 – 5.41 (m, 36H), 5.14 – 5.07 (m, 36H), 3.89 – 3.87 (m, 72H), 1.75 – 1.67 (m, 72H), 1.43 (m, 72H), 1.25 (m, 580H), 0.88 – 0.85 (m,108H),; ^{13}C NMR (125 MHz, $CDCl_3$ + DMSO-d6): δ ppm = 172.12, 164.47, 164.01, 157.98, 148.01, 147.91, 142.52, 135.18, 128.98, 113.52, 112.21, 111.42, 111.17, 106.27, 77.80, 73.59, 66.61, 66.37, 58.69, 58.30, 36.57, 35.05, 34.39, 34.36, 34.31, 34.10, 34.01, 30.84, 27.34, 18.97. Elemental analysis: C, 73.41; H, 9.91; N, 7.78 calculated (%): C, 73.69; H, 10.21; N, 7.51 (expt. %).

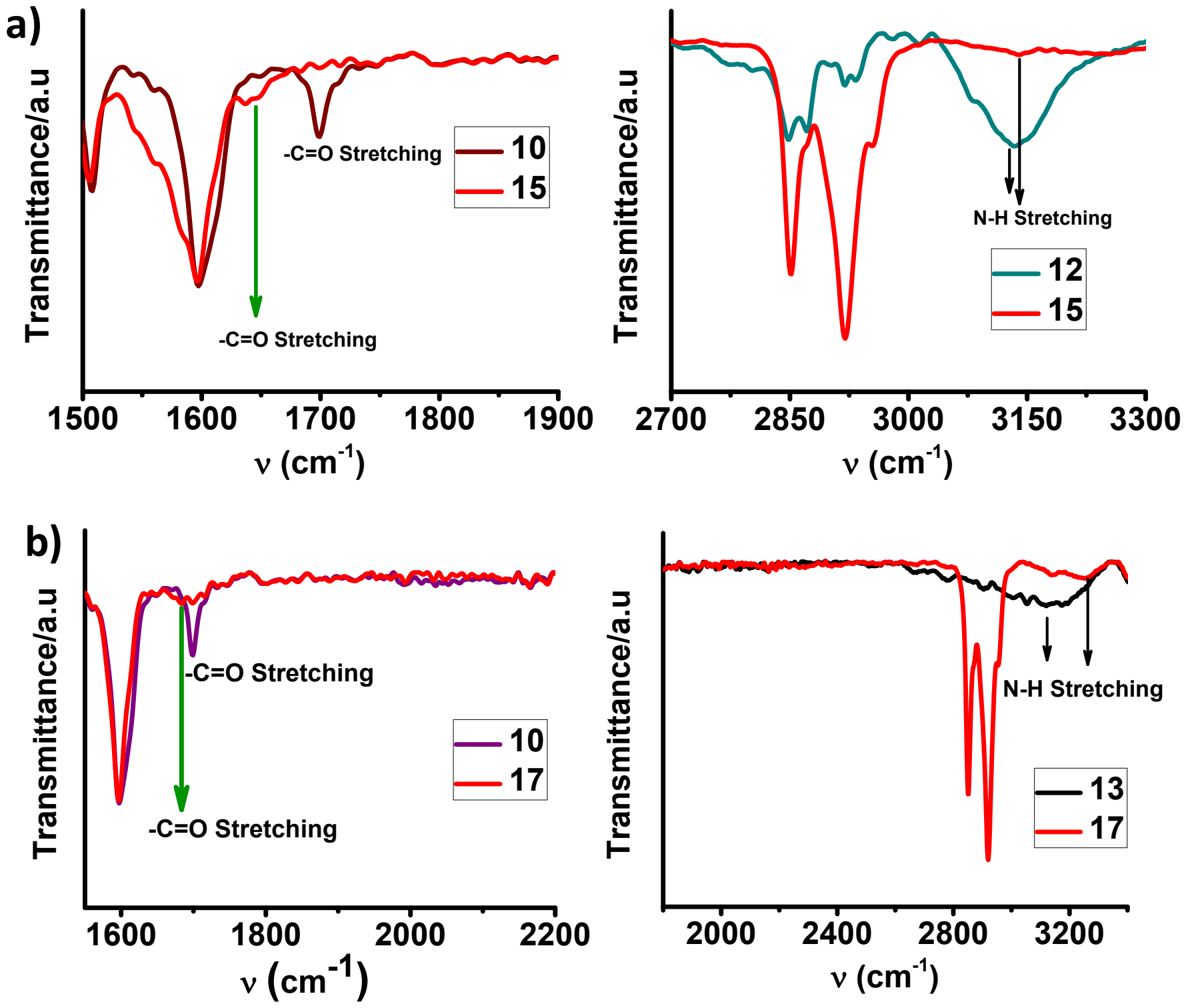

Figure S1: FTIR spectra: comparison in the –NH and C=O stretching region. **a)** Pure component of **10, 12** and its H-bonded complex of **15**. **b)** Pure component of **10, 13** and its H-bonded complex of **17**.

Table S1. IR stretching values of compounds **10 -17** in the region of –NH and the C=O stretching region.

Compound	C=O Stretching (cm^{-1})	N-H Stretching (cm^{-1})
**10**	1698	--
**11**	1696	--
**12**	--	3134
**13**	--	3129
**14**	1639	3239
**15**	1642	3140
**16**	1682	3250
**17**	1696	3258

Table S2: Observed and calculated d-spacings of the cubic phase (Pm3n) for compounds **9, 10, 14, 15, 16,** and **17.**

2θ	Observed d-spacing	hkl	Calculated d-spacing	Difference
**Compound 9**				
2.091	42.22	200	a= 84.44	
3.52	25.15	311	25.45	0.3
4.217	20.94	400	21.11	0.17
**Compound 10**				
2.033	43.421	200	a= 86.84	
2.284	38.654	210	38.83	0.18
2.504	35.253	211	35.45	0.2
3.696	23.883	320	24.08	0.2
3.827	23.05	321	23.20	0.15
4.089	21.589	400	21.71	0.13
**Compound 14**				
2.342	37.69	200	a= 75.38	
3.720	23.73	310	23.83	0.1
5.351	16.50	420	16.85	0.35
**Compound 15**				
1.878	46.967	200	a=93.93	
2.114	41.75	210	42.00	0.25
2.293	38.493	211	38.34	0.15
3.454	25.56	321	25.10	0.46
3.751	23.53	400	23.48	0.05
**Compound 16**				
2.176	40.561	200	a= 81.12	
2.426	36.360	210	36.27	0.09
2.661	33.180	211	33.118	0.07
4.077	21.65	321	21.68	0.03
4.366	20.22	400	20.28	0.06
5.849	15.09	521	14.81	0.28
**Compound 17**				
1.365	64.8659	200	a= 129.73	
1.903	46.16	220	45.86	0.3
2.139	41.27	310	41.02	0.25
2.339	37.74	222	37.45	0.29
2.715	32.51	400	32.43	0.08
3.028	29.15	420	29.00	0.15
3.312	26.65	422	26.48	0.17
3.461	25.50	500	25.94	0.44
3.572	24.69	511	24.99	0.43
3.828	23.06	440	22.93	0.13
4.658	18.95	631	18.92	0.03
4.879	18.09	711	18.16	0.07
5.165	17.09	722	17.18	0.09

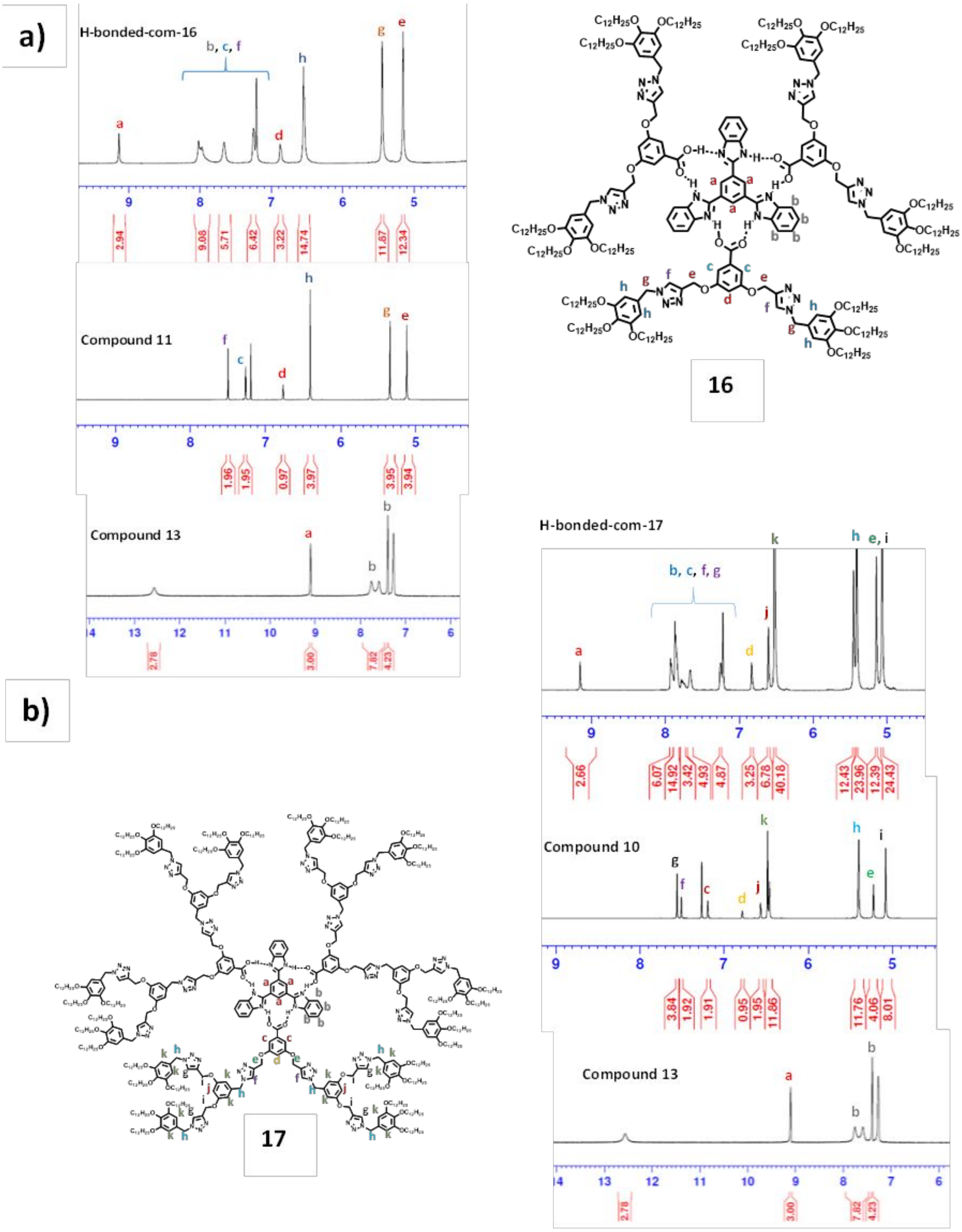

Figure S2. a) Comparative ^{1}H NMR studies of **11**, **13**, and the hydrogen complex of **16**. b) Comparative ^{1}H NMR studies of **10**, **13**, and the hydrogen complex of **17**.

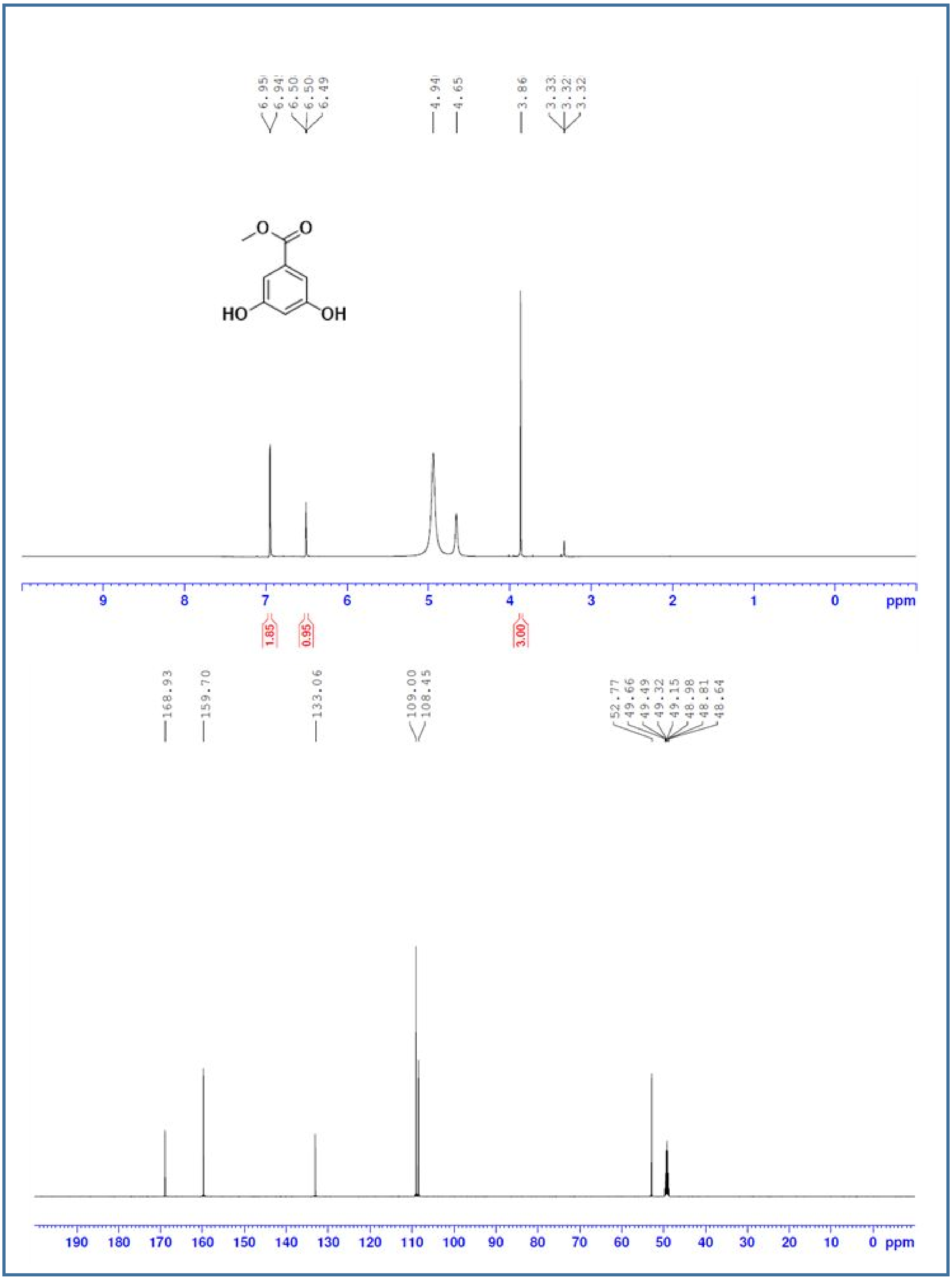

Figure S3: ^{1}H (top) and ^{13}C-NMR (bottom) spectra of **1**

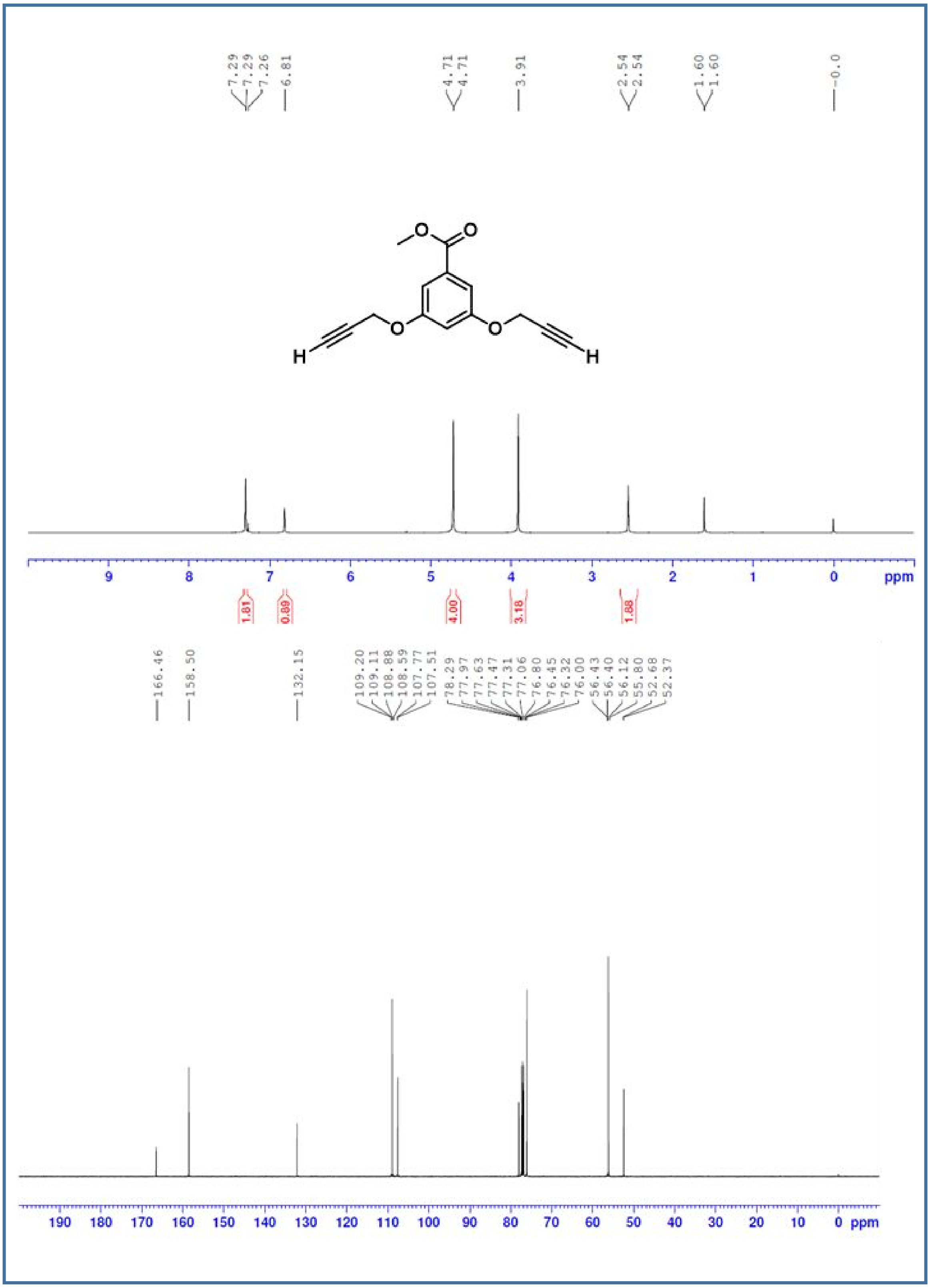

Figure S4: ^{1}H (top) and ^{13}C-NMR (bottom) spectra of **2**

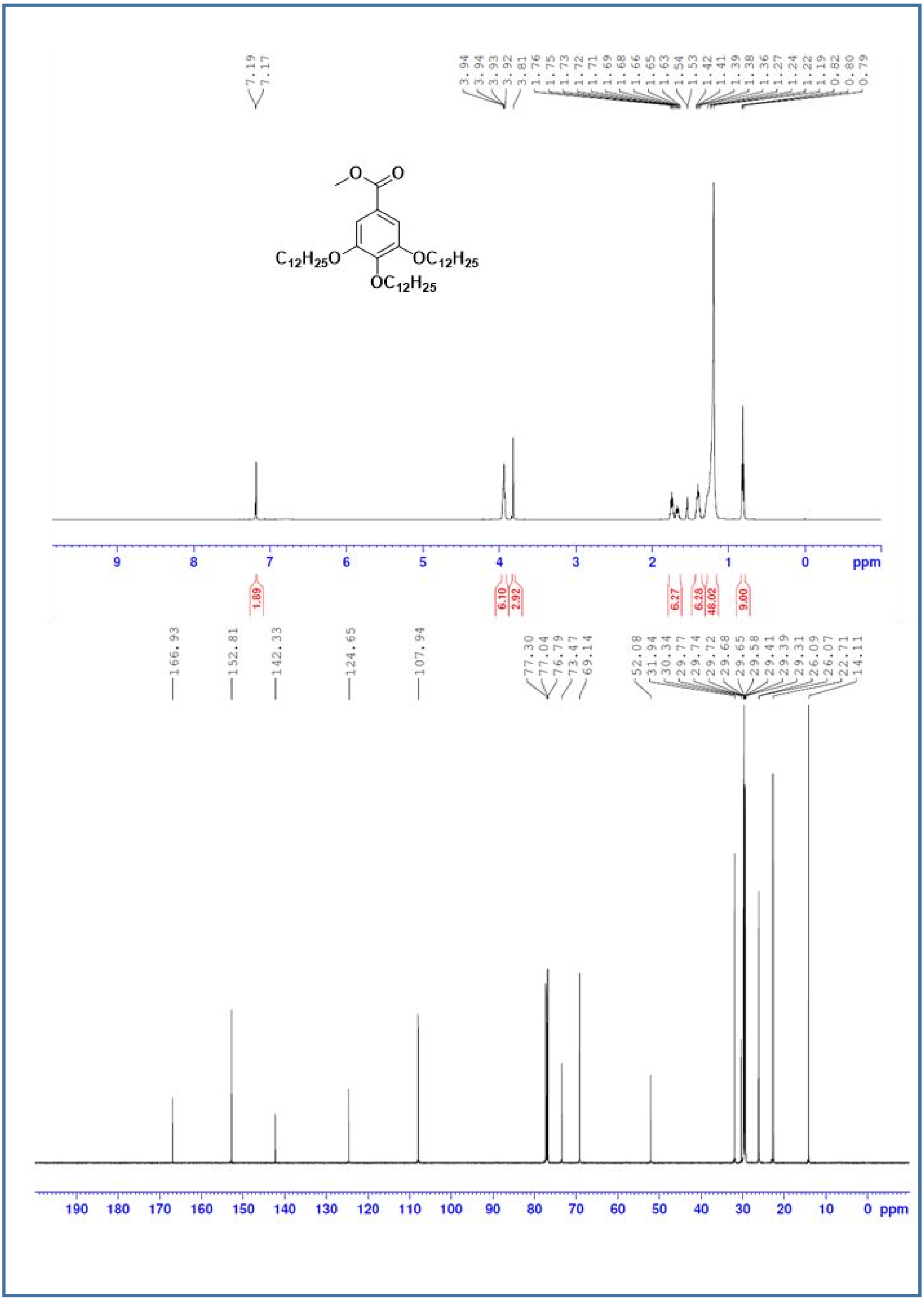

Figure S5: ^{1}H (top) and ^{13}C-NMR (bottom) spectra of **3**

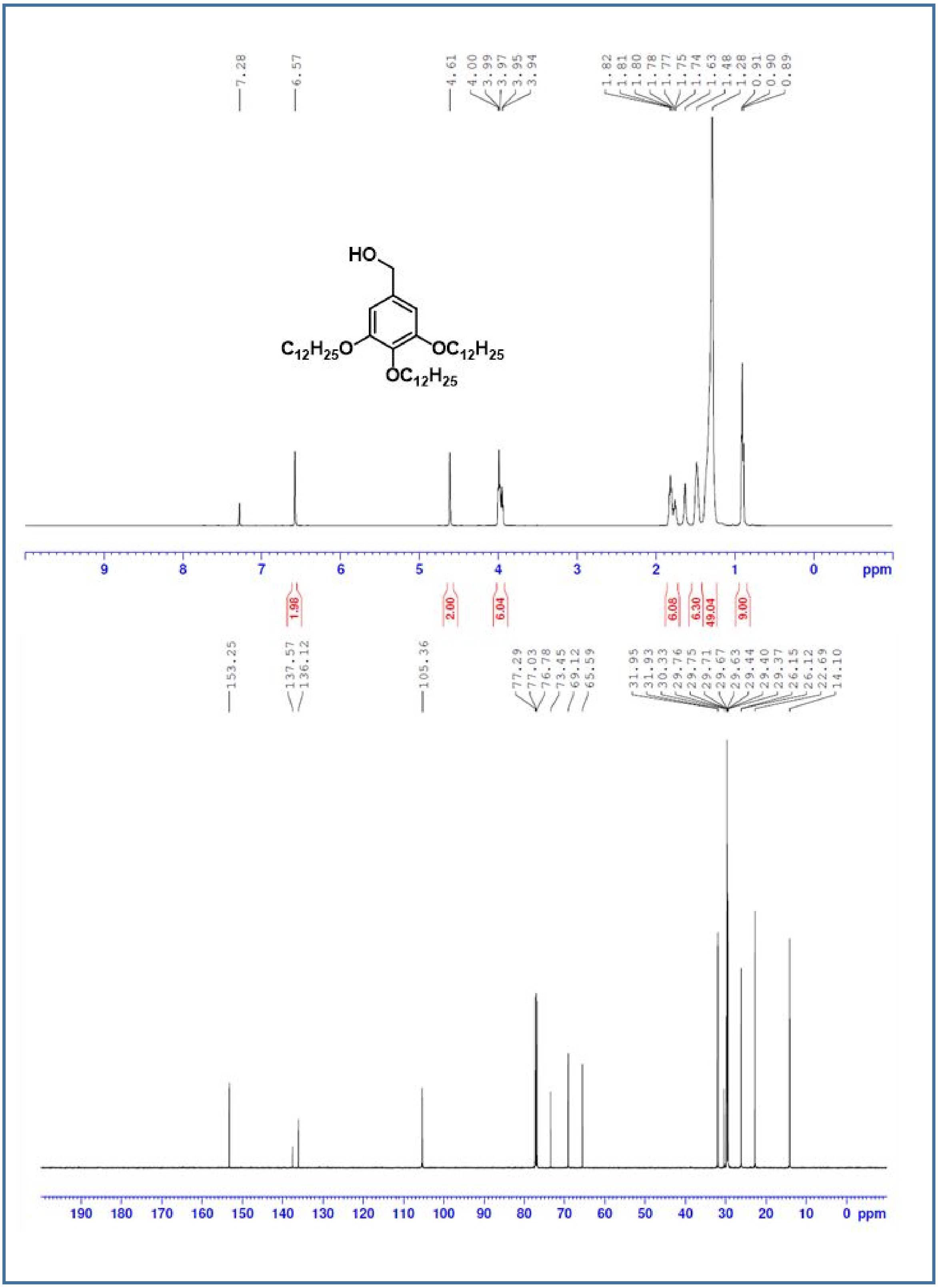

Figure S6: ^{1}H (top) and ^{13}C-NMR (bottom) spectra of **4**

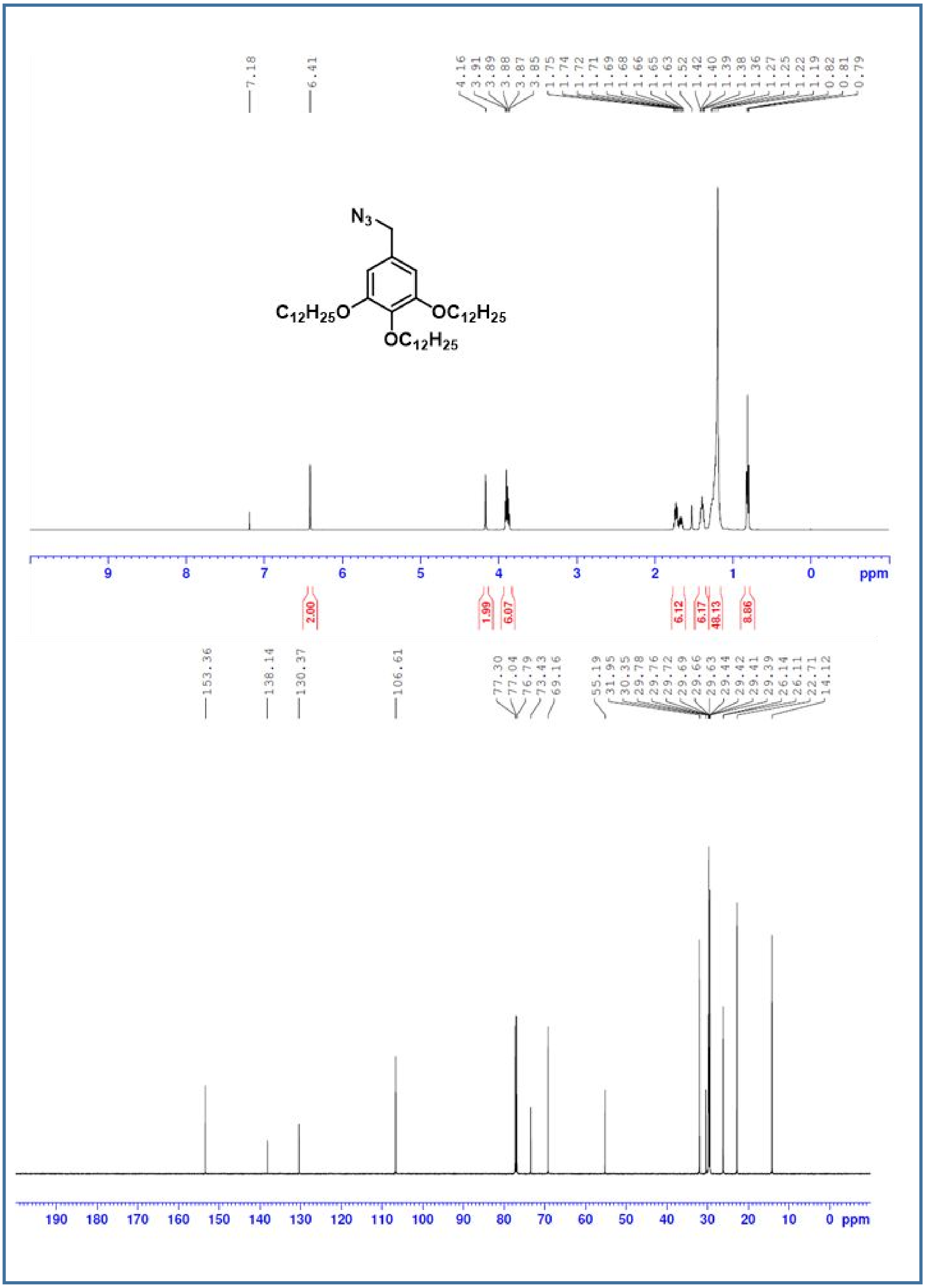

Figure S7: ^{1}H (top) and ^{13}C-NMR (bottom) spectra of **5**

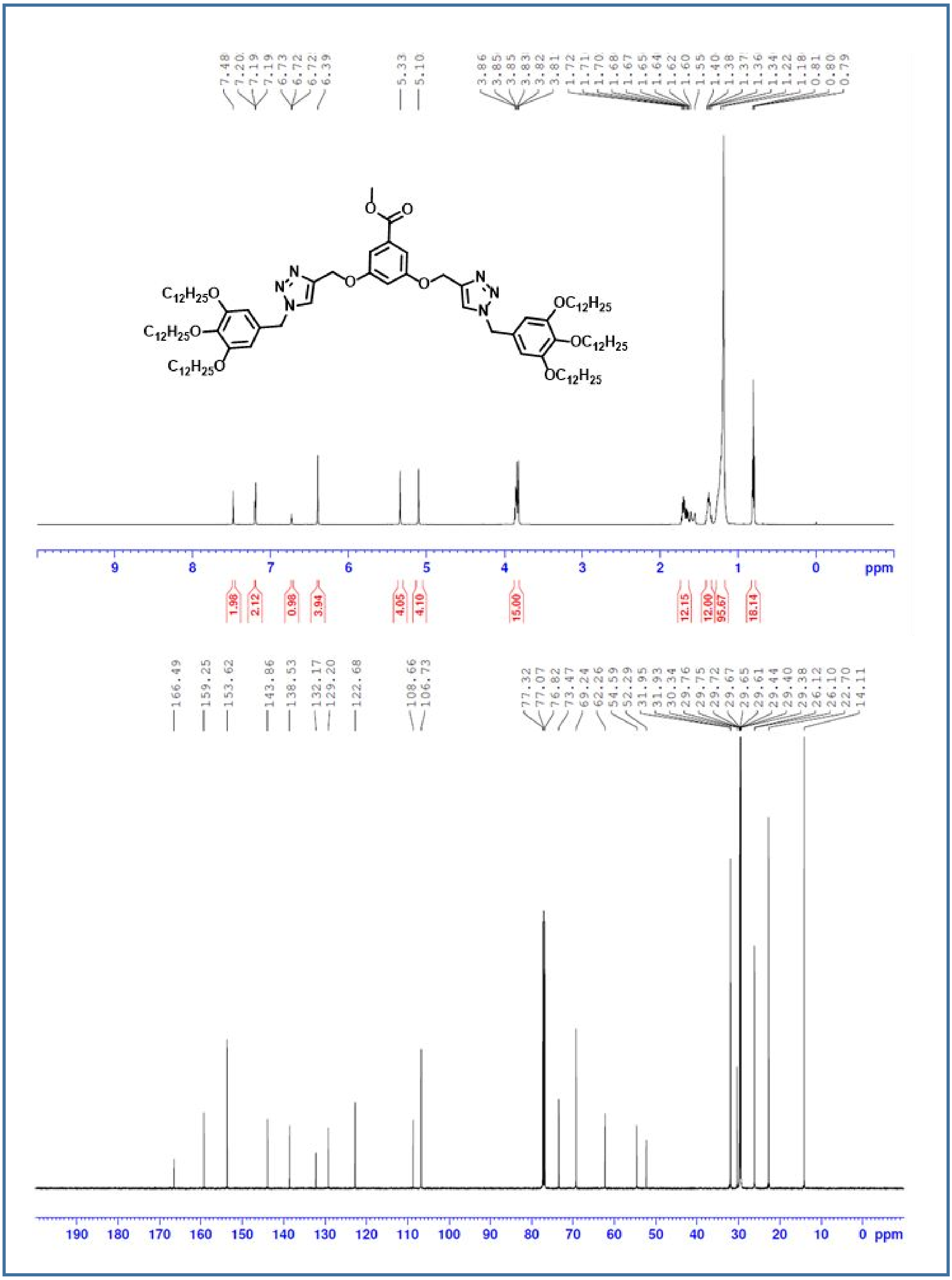

Figure S8: ^{1}H (top) and ^{13}C-NMR (bottom) spectra of **6**

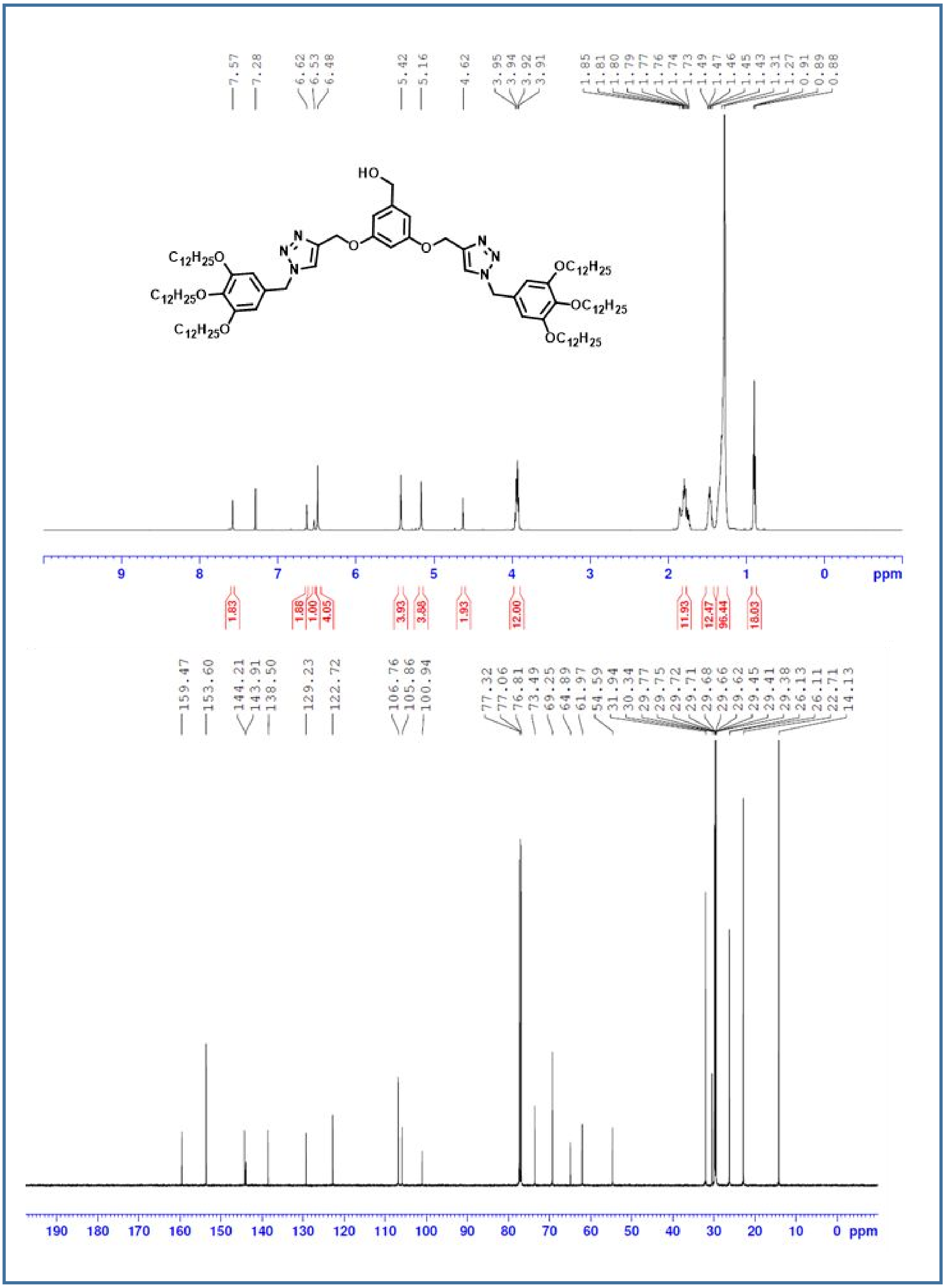

Figure S9: ^{1}H (top) and ^{13}C-NMR (bottom) spectra of **7**

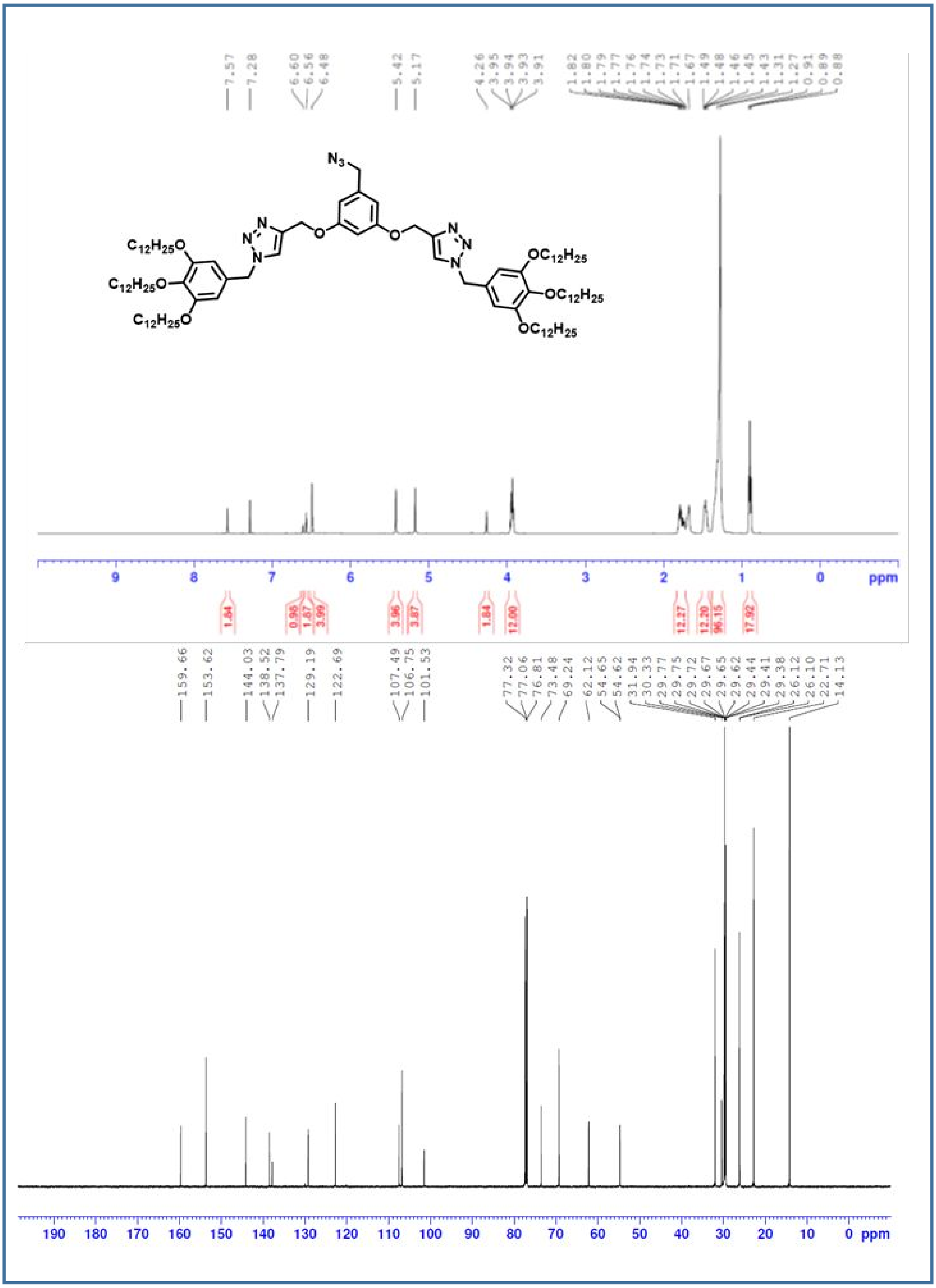

Figure S10: ^{1}H (top) and ^{13}C-NMR (bottom) spectra of **8**

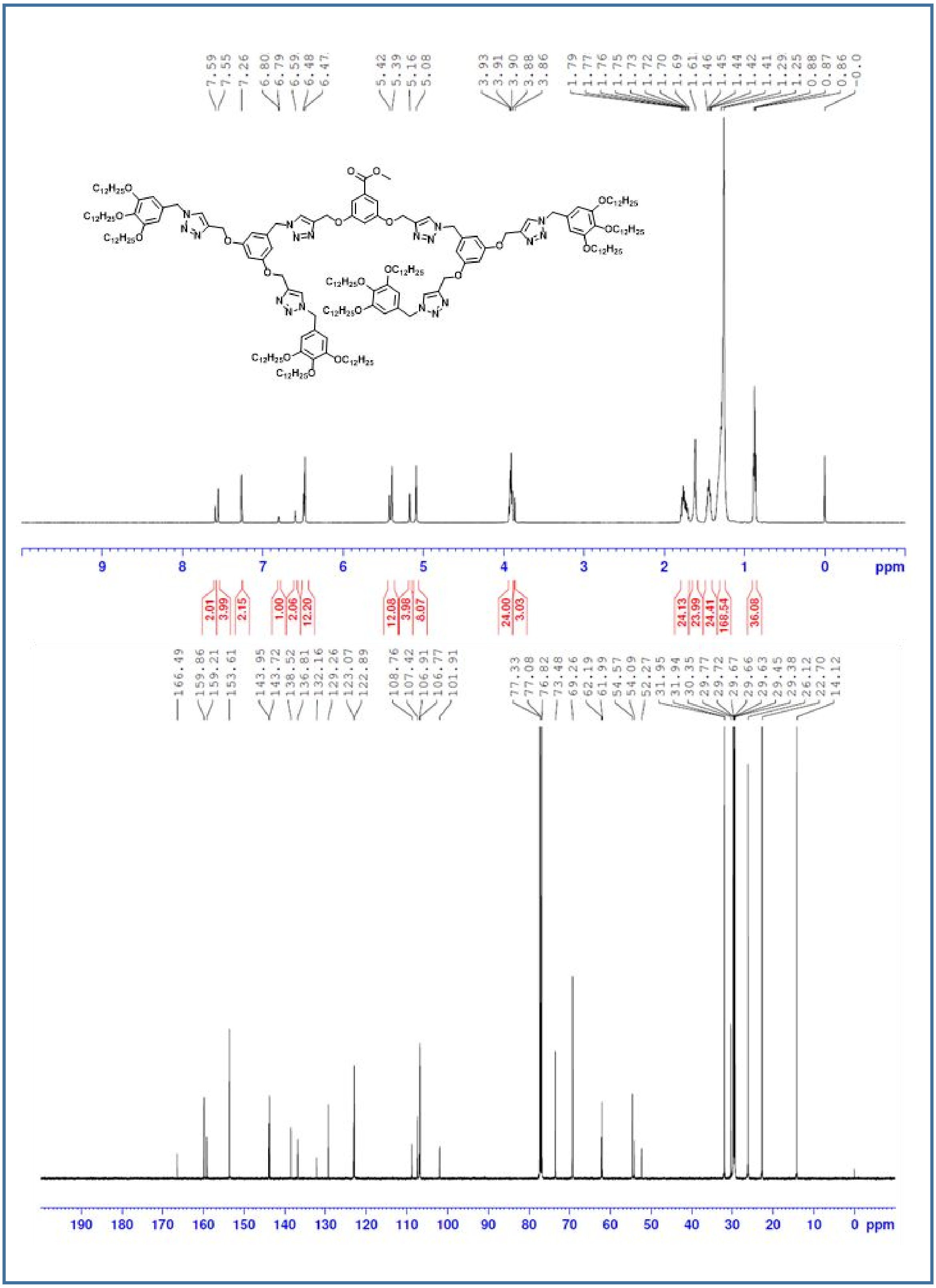

Figure S11: ^{1}H (top) and ^{13}C-NMR (bottom) spectra of **9**

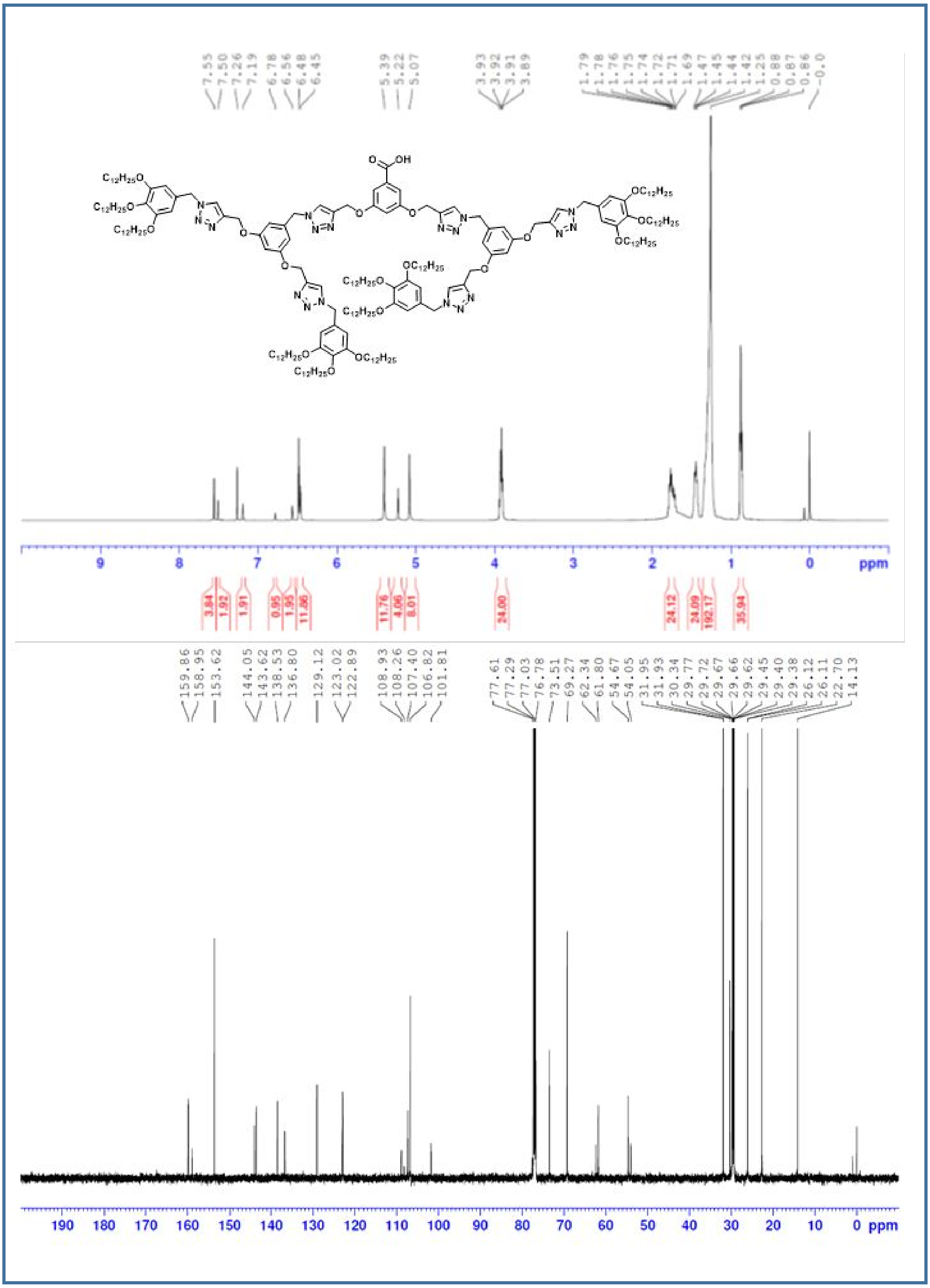

Figure S12: ^{1}H (top) and ^{13}C-NMR (bottom) spectra of **10**

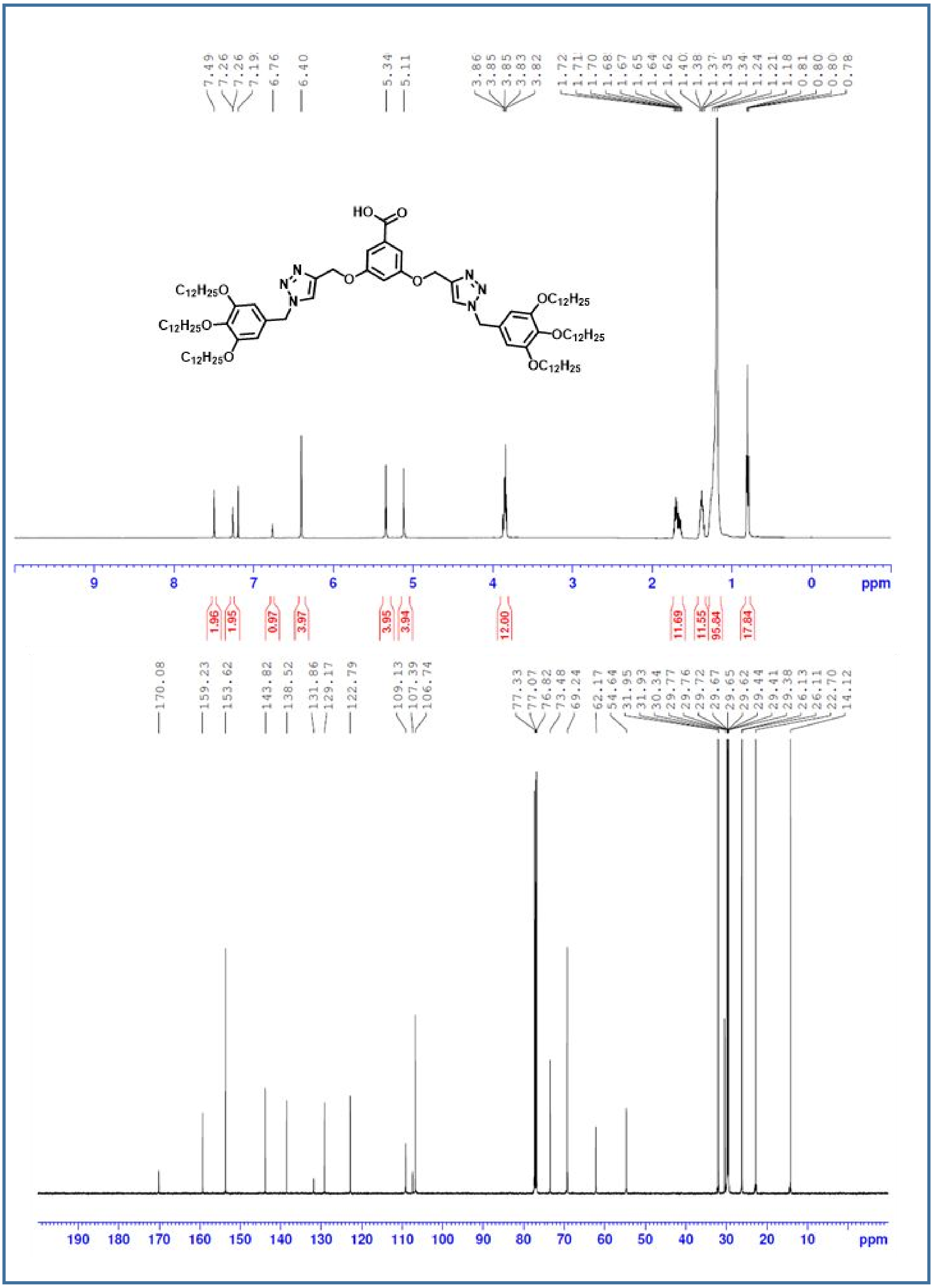

Figure S13: ^{1}H (top) and ^{13}C-NMR (bottom) spectra of **11**

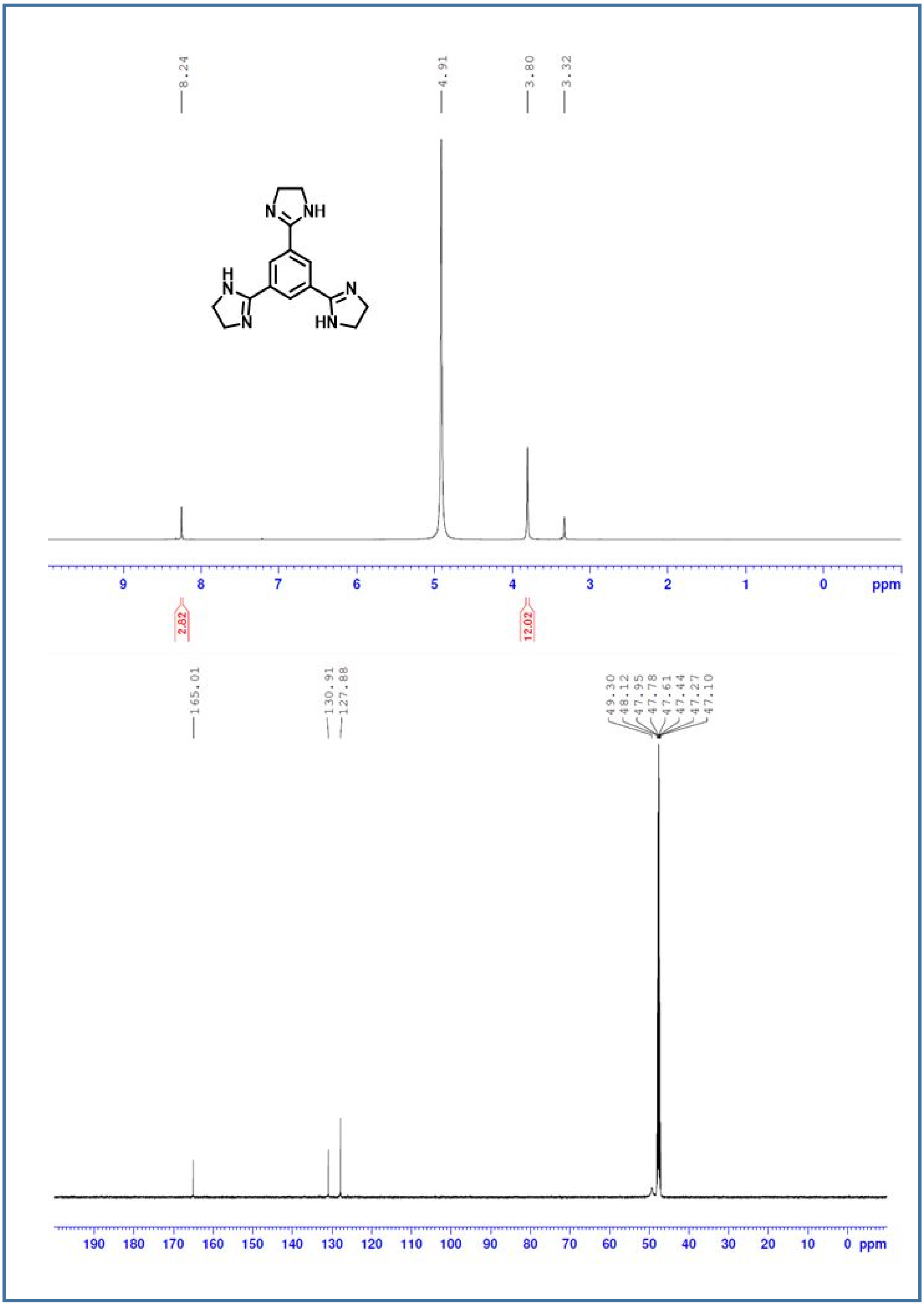

Figure S14: ^{1}H (top) and ^{13}C-NMR (bottom) spectra of **12**

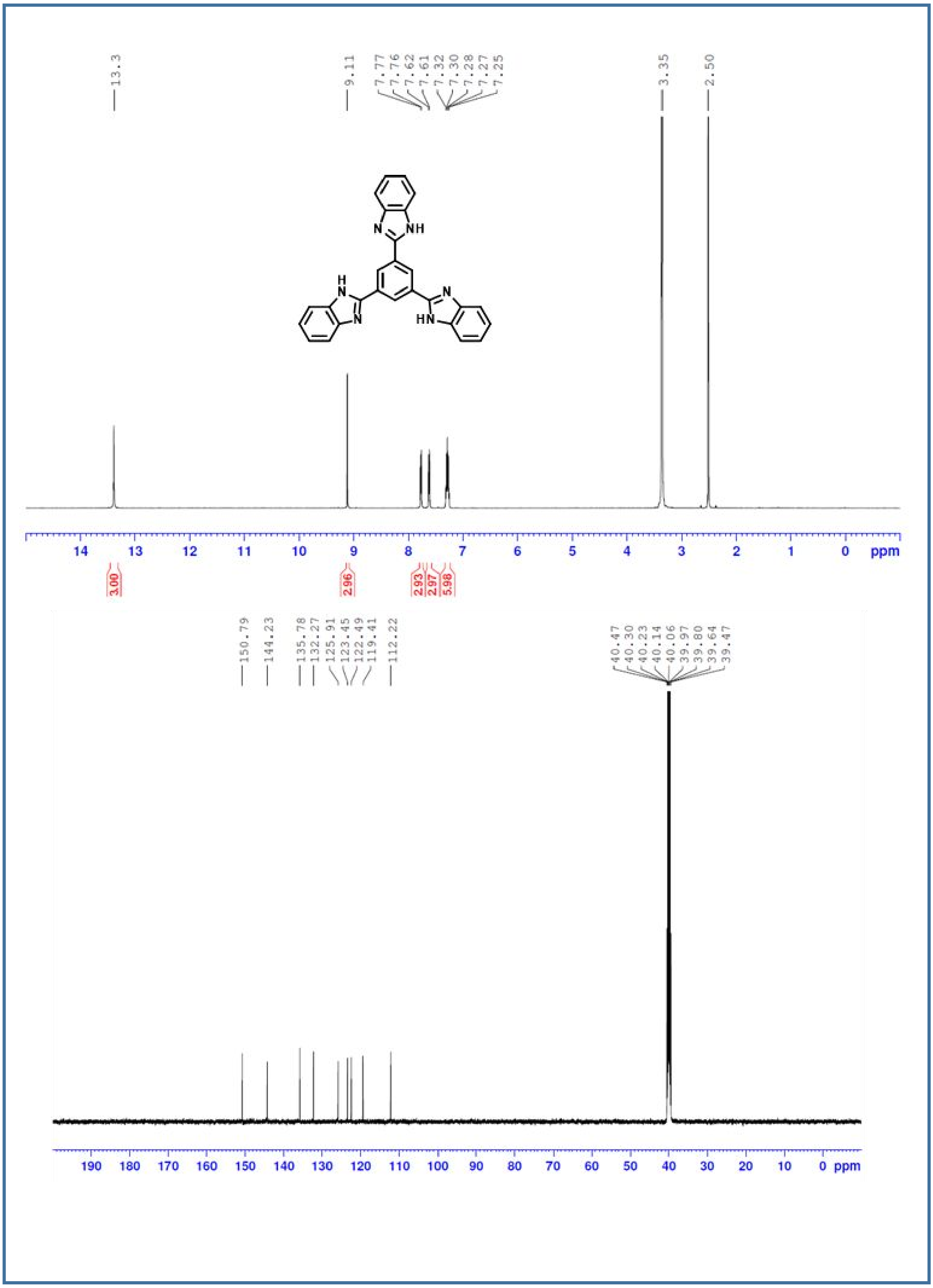

Figure S15: ^{1}H (top) and ^{13}C-NMR (bottom) spectra of **13**

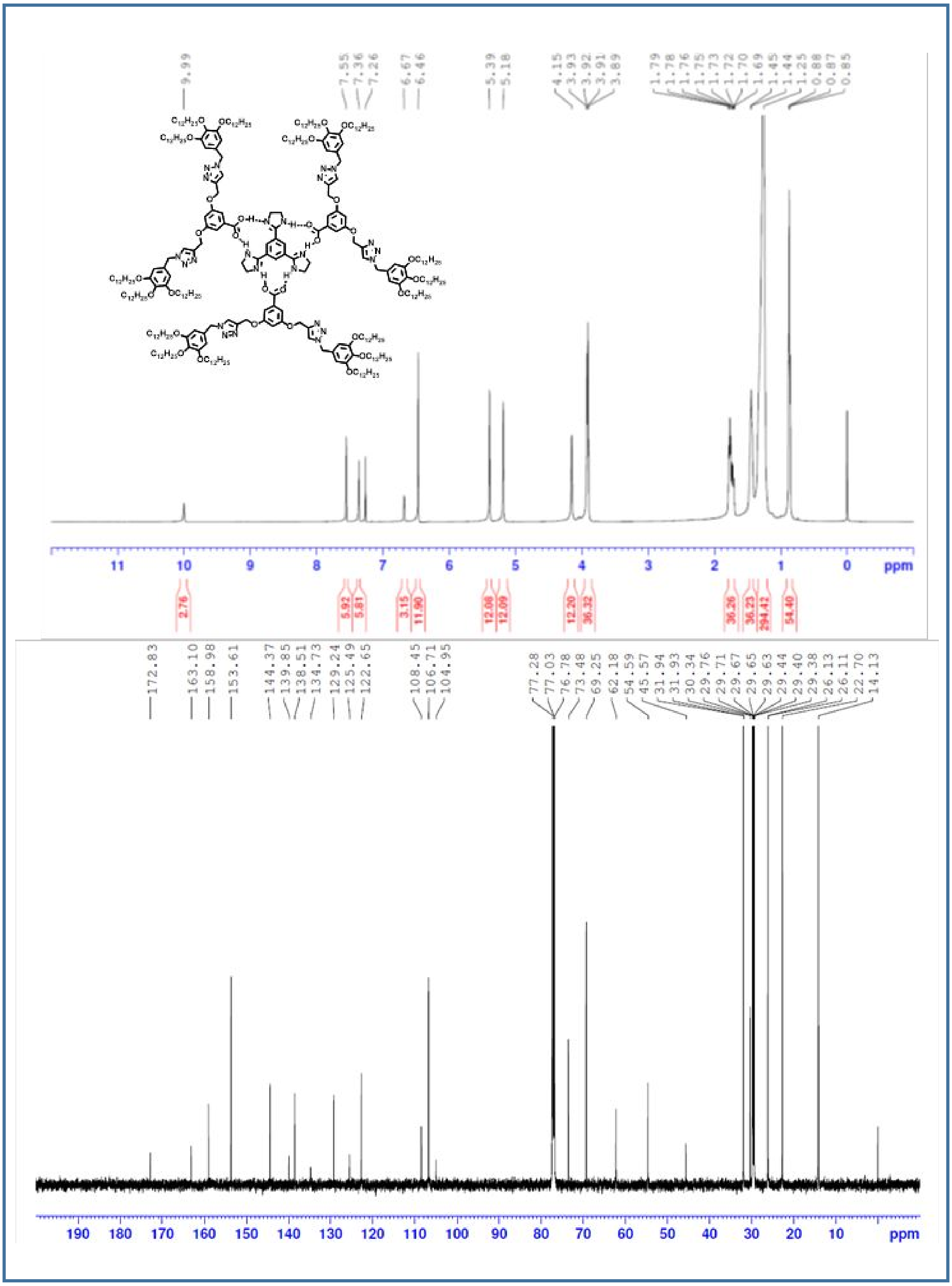

Figure S16: ^{1}H (top) and ^{13}C-NMR (bottom) spectra of **14**

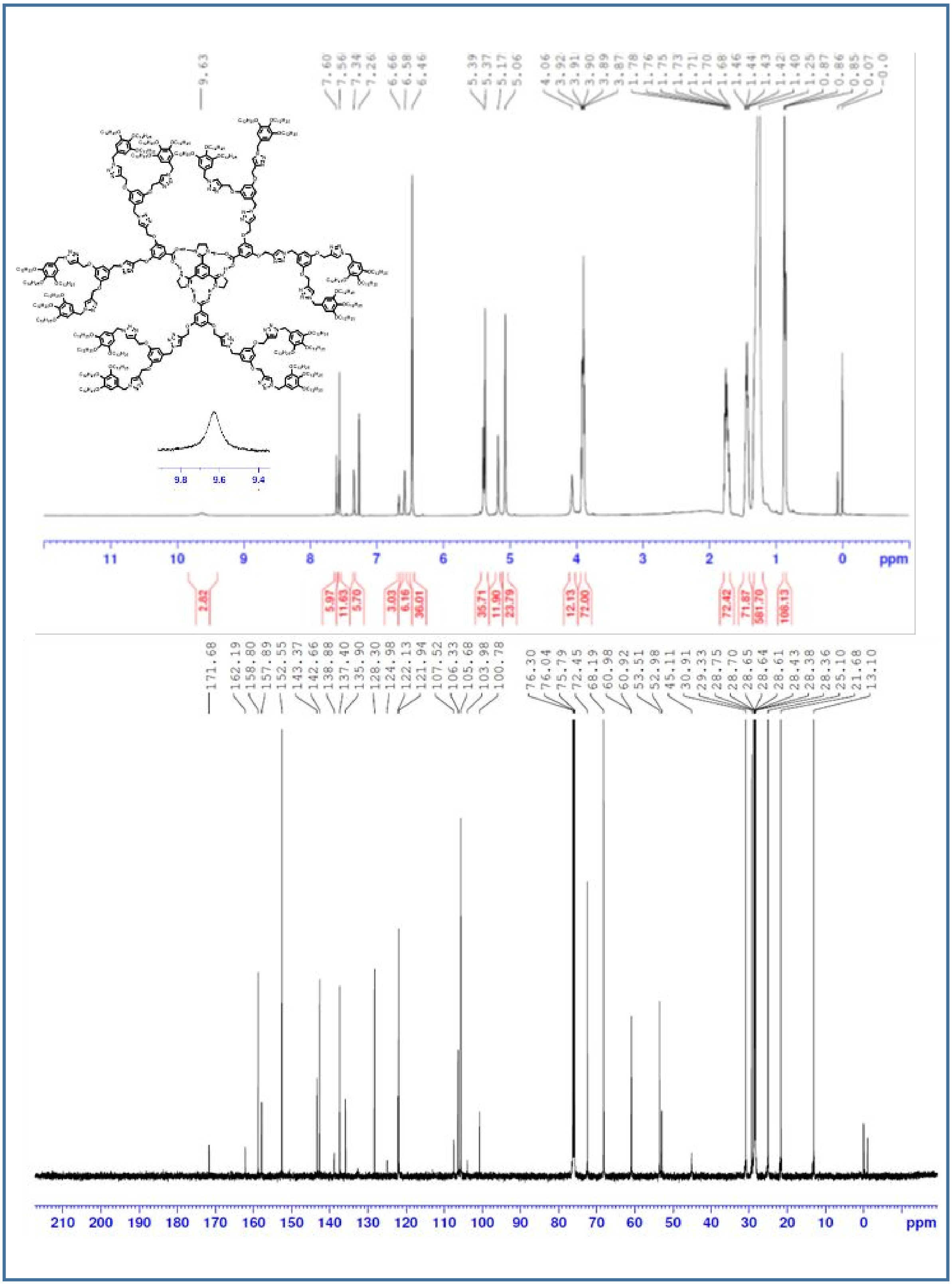

Figure S17: ^{1}H (top) and ^{13}C-NMR (bottom) spectra of **15**

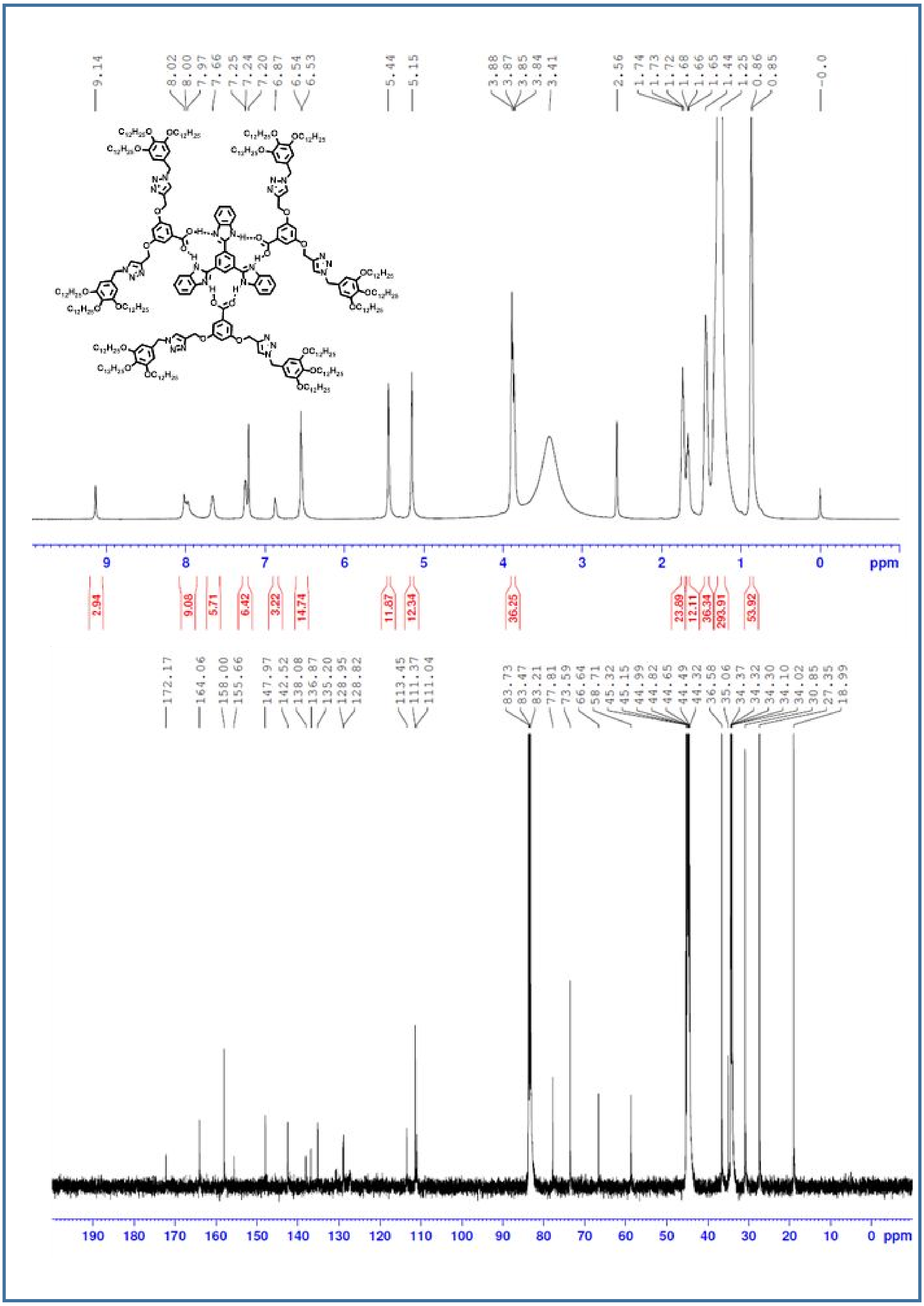

Figure S18: ^{1}H (top) and ^{13}C-NMR (bottom) spectra of **16**

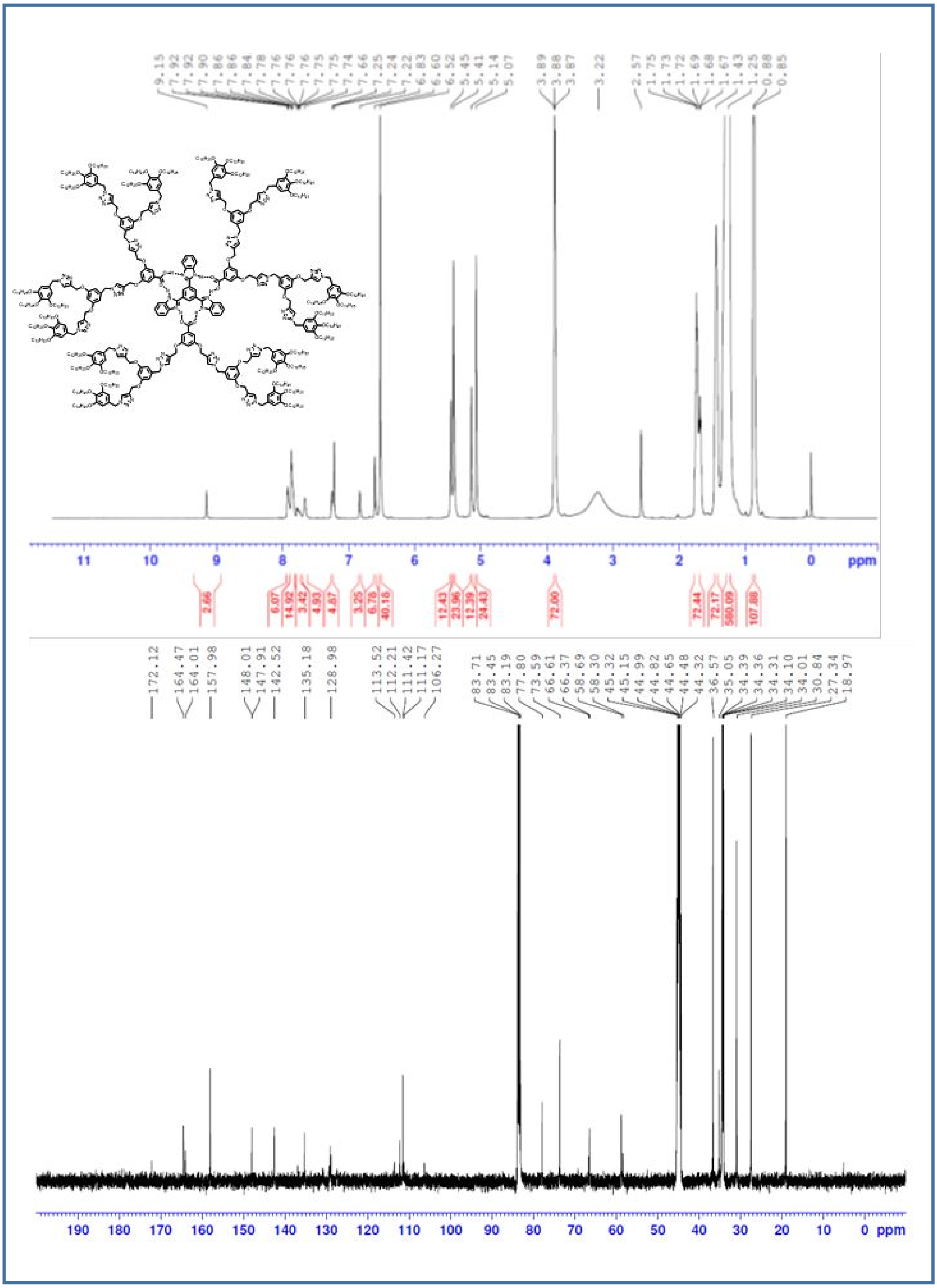

Figure S19: ^{1}H (top) and ^{13}C-NMR (bottom) spectra of **17**
